# Aging imparts cell-autonomous dysfunction to regulatory T cells during recovery from influenza pneumonia

**DOI:** 10.1172/jci.insight.141690

**Published:** 2021-03-22

**Authors:** Luisa Morales-Nebreda, Kathryn A. Helmin, Manuel A. Torres Acosta, Nikolay S. Markov, Jennifer Yuan-Shih Hu, Anthony M. Joudi, Raul Piseaux-Aillon, Hiam Abdala-Valencia, Yuliya Politanska, Benjamin D. Singer

**Affiliations:** 1 Department of Medicine, Division of Pulmonary and Critical Care Medicine,; 2 Department of Biochemistry and Molecular Genetics, and; 3 Simpson Querrey Institute for Epigenetics, Northwestern University Feinberg School of Medicine, Chicago, Illinois, USA.

**Keywords:** Aging, T cells

## Abstract

Regulatory T (Treg) cells orchestrate resolution and repair of acute lung inflammation and injury after viral pneumonia. Compared with younger patients, older individuals experience impaired recovery and worse clinical outcomes after severe viral infections, including influenza and SARS coronavirus 2 (SARS-CoV-2). Whether age is a key determinant of Treg cell prorepair function after lung injury remains unknown. Here, we showed that aging results in a cell-autonomous impairment of reparative Treg cell function after experimental influenza pneumonia. Transcriptional and DNA methylation profiling of sorted Treg cells provided insight into the mechanisms underlying their age-related dysfunction, with Treg cells from aged mice demonstrating both loss of reparative programs and gain of maladaptive programs. Strategies to restore youthful Treg cell functional programs could be leveraged as therapies to improve outcomes among older individuals with severe viral pneumonia.

## Graphical abstract

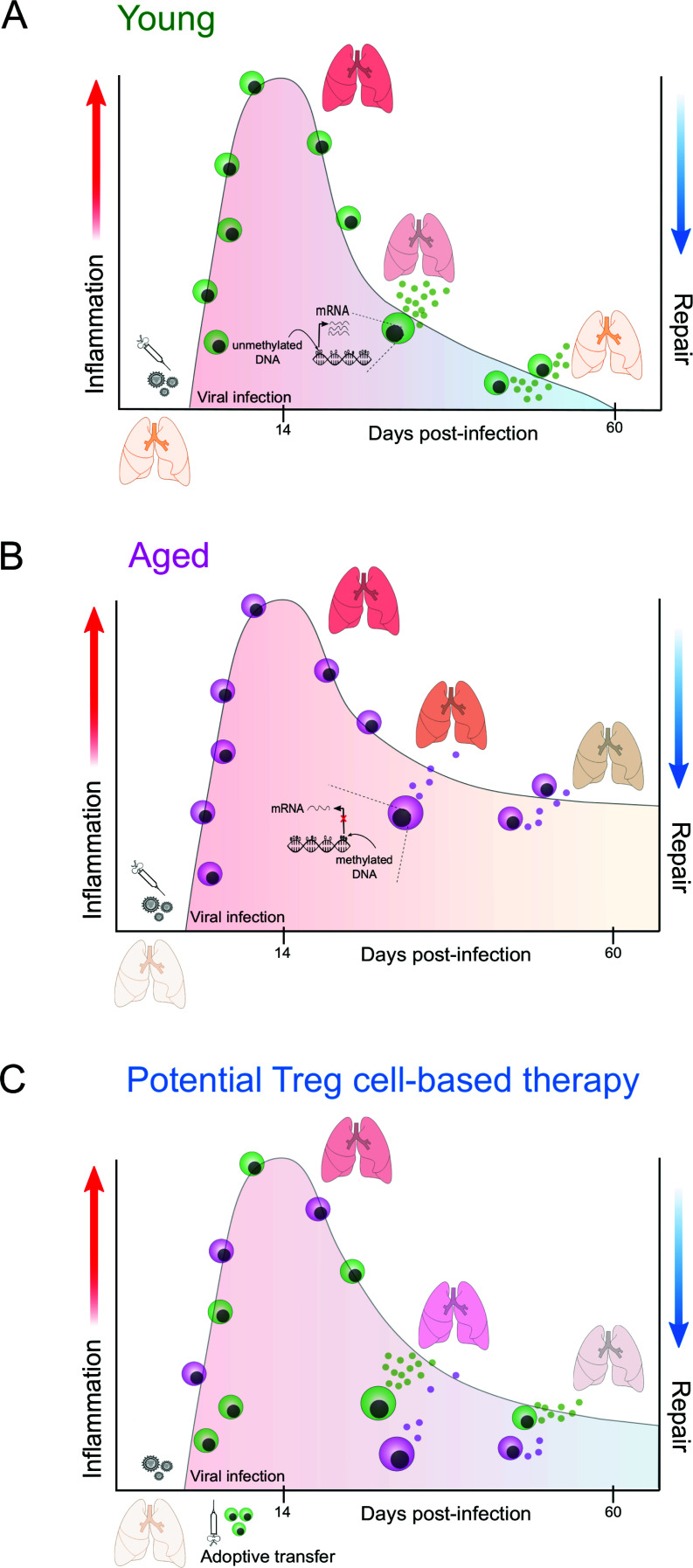

## Introduction

Age is the most important risk factor determining mortality and disease severity in patients infected with influenza virus or SARS coronavirus 2 (SARS-CoV-2; refs. 1–3). Global estimates of seasonal influenza-associated mortality range from 300,000 to 650,000 deaths per year, with the highest at-risk group consisting of individuals over age 75 (4). In the United States, influenza-associated morbidity and mortality have steadily increased, an observation linked to an expansion of the aging population. Pneumonia related to severe influenza A virus and SARS-CoV-2 infection results in an initial acute exudative phase characterized by release of proinflammatory mediators that damage the alveolar epithelial and capillary barrier to cause refractory hypoxemia and acute respiratory distress syndrome (ARDS) (5). If a patient survives this first stage, activation of resolution and repair programs during the ensuing recovery phase is crucial for restoration of lung architecture and function, which promotes liberation from mechanical ventilation, decreases intensive care unit length of stay, and extends survival.

Immunomodulatory regulatory T (Treg) cells expressing the lineage-specifying transcription factor Foxp3 dampen inflammatory responses to endogenous and exogenous antigens. Aside from their role in maintaining immune homeostasis through their capacity to suppress overexuberant immune system activation, Treg cells reside in healthy tissues and accumulate in the lung in response to viral injury to promote tissue repair (6–8). Our group and others have shown that in murine models of lung injury, Treg cells are master orchestrators of recovery (9–12). Treg cells are capable of promoting tissue regeneration and repair, at least in part through release of reparative mediators, such as the EGF receptor ligand amphiregulin (Areg), which induces cell proliferation and differentiation of the injured tissue (13).

Epigenetic phenomena, including DNA methylation, modify the architecture of the genome to control gene expression and regulate cellular identity and function throughout the lifespan (14). Aside from being one of the best predictive biomarkers of chronological aging and age-related disease onset, DNA methylation regulates Treg cell identity through tight epigenetic control of *Foxp3* and Foxp3-dependent programs (15). Biological aging is associated with a progressive loss of molecular and cellular homeostatic mechanisms that maintain normal organ function, rendering individuals susceptible to disease (16, 17). Because of their tissue-reparative functions, Treg cells are important modulators of the immune response that promotes tissue regeneration after injury (18). Whether age plays a key role in determining the prorepair function of Treg cells in the injured lung during recovery from viral pneumonia remains unknown. If aging indeed affects Treg cell–mediated recovery, is it a Treg cell–autonomous phenomenon or is it because the aging lung microenvironment is resistant to Treg cell–mediated repair? Using heterochronic (age-mismatched) adoptive Treg cell transfer experiments and molecular profiling in mice, we sought to determine whether the age-related impairment in repair after influenza-induced lung injury is intrinsic to Treg cells. Our data support a paradigm in which aged Treg cells activate maladaptive responses, fail to upregulate youthful reparative programs, and consequently exhibit a cell-autonomous impairment in prorecovery function, which delays resolution from virus-induced lung injury among aged hosts.

## Results

### Aging results in increased susceptibility to influenza-induced lung injury because of impaired recovery.

**Figure 1:**
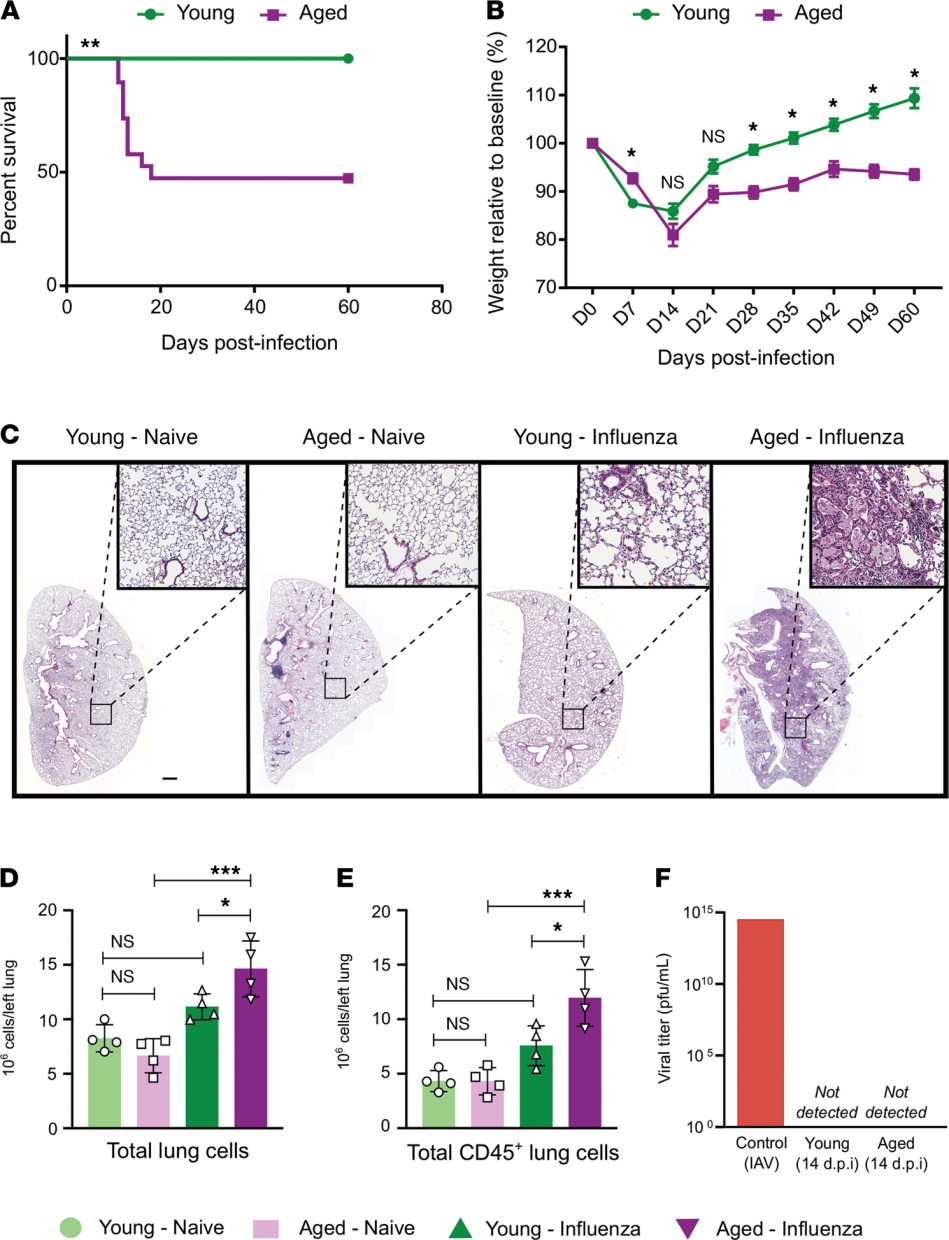
Aged mice demonstrate increased mortality and lung inflammation during recovery from influenza infection. (**A**) Survival curve comparison of young (2 months, *n* = 20) and aged (18 months, *n* = 19) WT mice using log-rank (Mantel-Cox) test. (**B**) Weight loss percentage from baseline in young (2–4 months, *n* = 20) and aged (18–22 months, *n* = 19) mice compared using a mixed-effects model (REML) with Sidak’s post hoc multiple-comparison test. Data presented as mean ± SEM. (**C**) Representative lung histopathology (H&E staining) of young and aged mice during the naive state and recovery phase after influenza infection (day 60). Original magnification ×10. Scale bar: 500 μm. (**D**) Flow cytometry quantitative analysis of total number of cells and (**E**) total number of CD45^+^ cells from left lung during the naive state and recovery phase from influenza infection. (**F**) Viral titer measurement of lung homogenates in young and aged mice 14 days after infection. Positive control from influenza A/WSN/33 (H1N1) viral stock. *n* = 4 mice/group (young — influenza and aged — influenza). Data presented as mean ± SD, 1-way ANOVA with Holm-Sidak’s post hoc testing for multiple comparisons (**D** and **E**). *P < 0.05; **P < 0.005; ***P < 0.0005. NS, not significant. *n* = 4 mice/group (young — naive, aged — naive, young — influenza, and aged — influenza) for **D** and **E**.

To evaluate the age-related susceptibility to influenza-induced lung injury, we administered influenza A/WSN/33 (H1N1) virus via the intratracheal route to young (2–4 months) and aged (18–22 months) WT mice. Aged mice exhibited greater than 50% mortality when compared with young animals (Figure 1A), impaired recovery of total body weight following a similar nadir (Figure 1B), and more severe lung injury by histopathology at a late recovery time point, day 60 after infection (Figure 1C). At this same time point, aged mice also displayed an increase in the total number of cells per lung (Figure 1D), which were mainly composed of immune cells identified by the pan-hematopoietic marker CD45 (Figure 1E), suggesting nonresolving tissue inflammation during recovery in older mice. Similar to prior reports (19, 20), we confirmed that the increased susceptibility to influenza-induced lung injury in aged mice was observed despite having cleared the virus on day 14 after infection, the time of the greatest degree of weight loss among both groups (Figure 1F).

We next wanted to determine whether the age-related susceptibility to influenza-induced lung injury was due to a differential inflammatory response during the initial acute injury phase. Accordingly, we examined a different group of young and aged mice at a time point when viral clearance was complete and weight nadir was observed in both groups, 14 days after infection. Aged mice demonstrated increased mortality when compared with young animals at this time point (Supplemental Figure 1A; supplemental material available online with this article; https://doi.org/10.1172/jci.insight.141690DS1), but other markers of acute inflammation, including weight loss (Supplemental Figure 1B), total lung cells (Supplemental Figure 1C), and total lung CD45^+^ cells in surviving animals were not significantly different between groups (Supplemental Figure 1D). Collectively, these results suggest that aging results in similar early injury but persistent lung inflammatory pathology during the recovery phase of influenza-induced lung injury.

### Aging results in deficient repair after influenza-induced lung injury.

**Figure 2:**
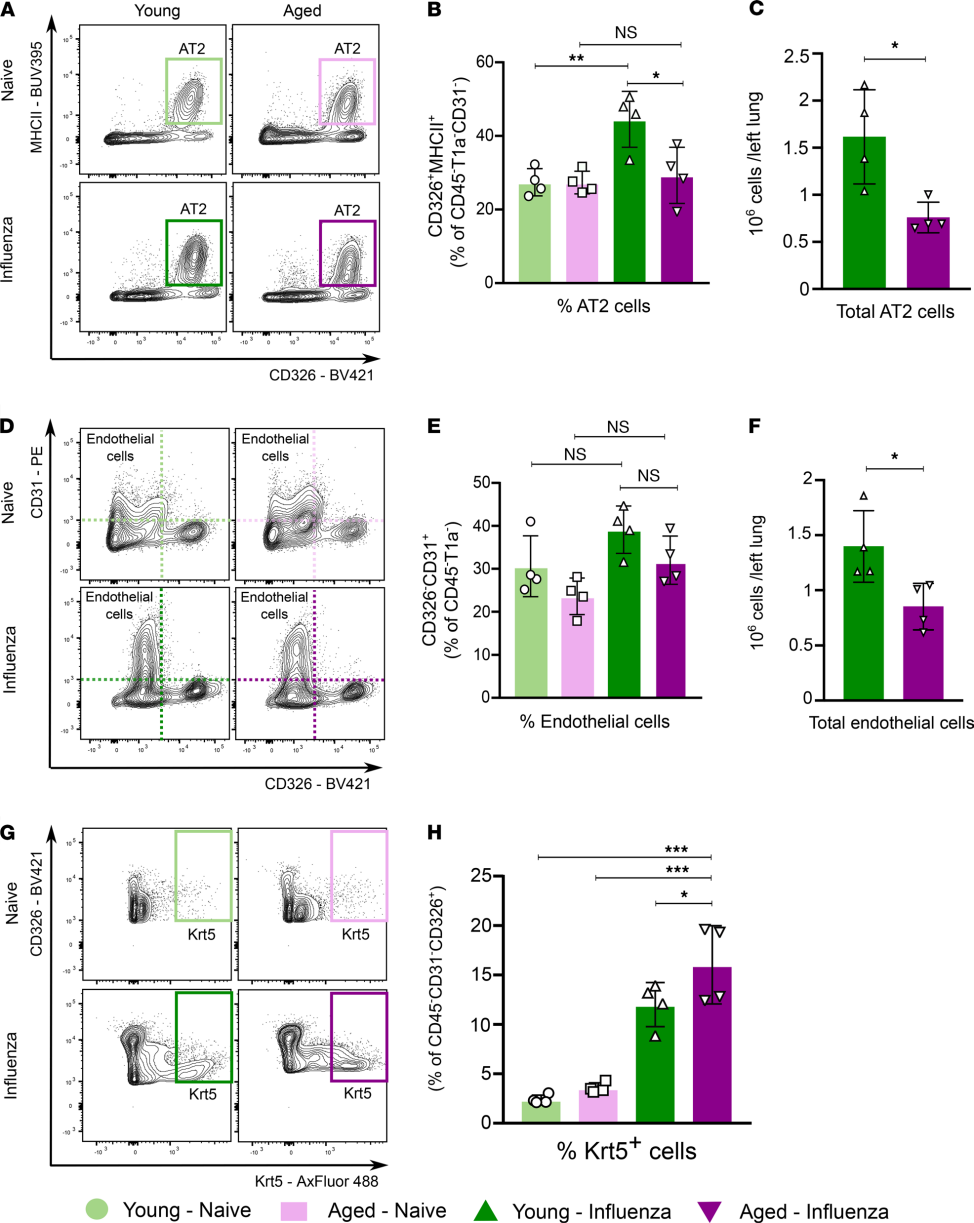
Aging results in failure to repopulate the alveolar epithelial-capillary barrier during recovery 60 days after influenza infection. (**A**) Representative flow cytometry contour plot analysis of type II alveolar epithelial cells (AT2). See Supplemental Figure 2 for parent gating. (**B**) CD326^+^MHCII^+^ type II alveolar epithelial cells as a percentage of CD45^–^T1α^–^CD31^–^ cells. (**C**) Total type II alveolar epithelial cells from left lung. (**D**) Representative flow cytometry contour plot analysis of endothelial cells. (**E**) CD326^–^CD31^+^ endothelial cells as a percentage of CD45^–^T1α^–^ cells. (**F**) Total endothelial cells from left lung. (**G**) Representative flow cytometry contour plot analysis of Krt5^+^ cells. (**H**) Krt5^+^ epithelial cells as a percentage of CD45^–^CD31^–^CD326^+^ cells. Data presented as mean ± SD, 1-way ANOVA with Holm-Sidak’s post hoc testing for multiple comparisons (**B**, **E**, and **H**) or Mann-Whitney test (**C** and **F**). *P < 0.05; **P < 0.005; ***P < 0.0005. NS, not significant. *n* = 4 mice/group (young — naive, aged — naive, young — influenza, and aged — influenza) for all panels.

Having established that aging results in an increased susceptibility to persistent lung inflammation and injury after influenza infection, we explored whether the impaired recovery in aged mice was linked to a persistent failure to repopulate the structural components of the alveolar-capillary barrier (i.e., failure to repair). Flow cytometry analysis (Supplemental Figure 2) of lung single-cell suspensions on day 60 after influenza infection revealed an increased percentage of alveolar epithelial type 2 (AT2) cells (CD45^–^T1α^–^CD31^–^EpCAM^+^/CD326^+^MHCII^+^) and endothelial cells (CD45^–^T1α^–^EpCAM^–^/CD326^–^CD31^+^) (Figure 2, A and B, and Figure 2, D and E, respectively) compared with the naive state. Compared with young animals, aged mice after influenza displayed a significantly lower total number of AT2 and endothelial cells (Figure 2C and Figure 2F, respectively).

In previous studies, investigators demonstrated that after influenza-induced lung injury, a population of cytokeratin 5^+^ (Krt5^+^) basal-like cells expand and migrate to the distal airspaces in an attempt to repair the injured epithelial barrier (21). These cells lack the capacity to transdifferentiate into functional AT2 cells, resulting in a dysplastic response that contributes to a dysregulated and incomplete repair phenotype after injury (22). Using a flow cytometry quantitative approach, we found that at 60 days after infection, aged mice exhibited a significant increase in Krt5^+^ cells compared with young animals (Figure 2, G and H). In summary, older mice failed to repair the injured lung during the recovery phase of influenza-induced lung injury.

### Aging determines the prorecovery function of Treg cells after influenza-induced lung injury.

**Figure 3:**
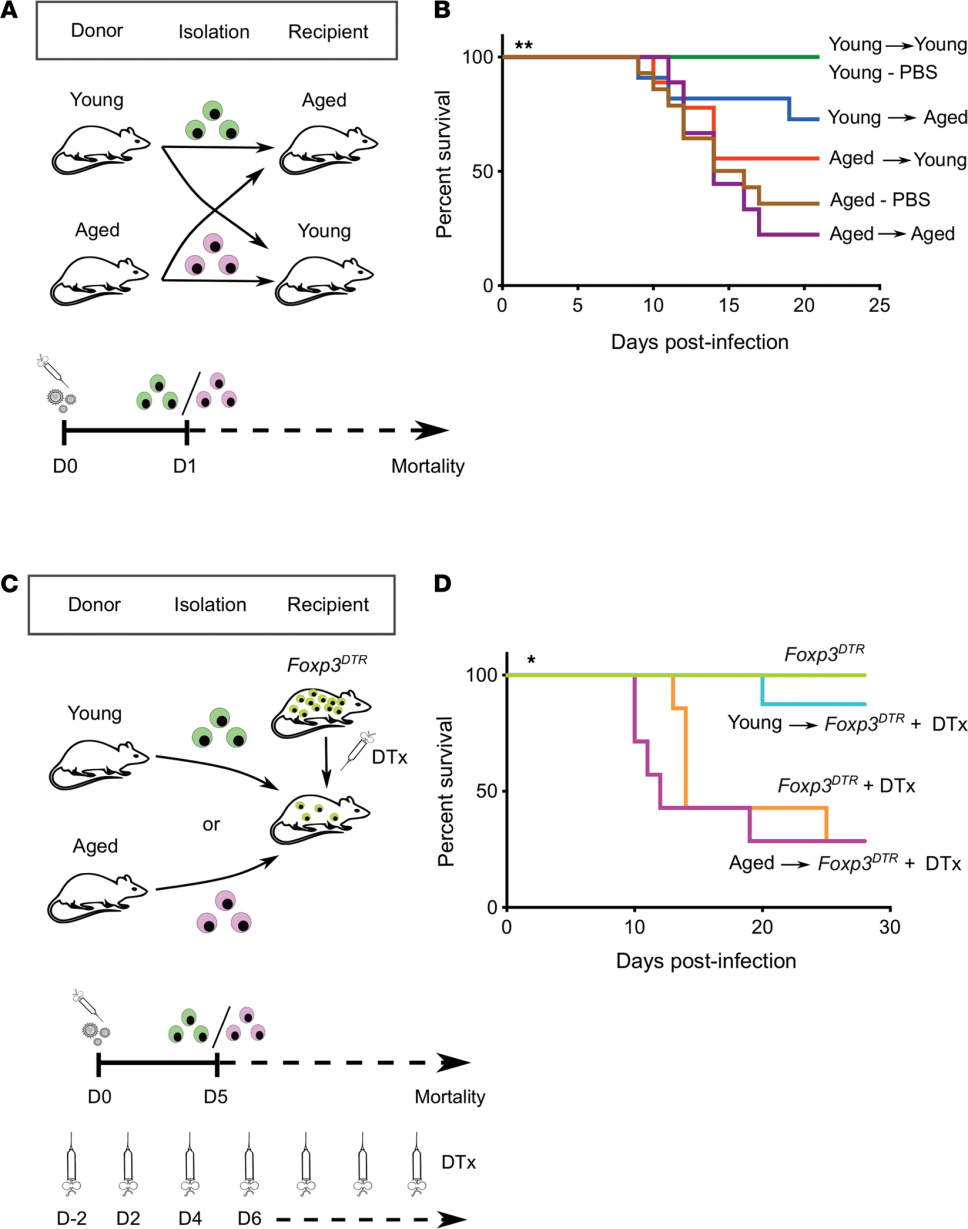
Age determines the tissue-protective phenotype of Treg cells during influenza-induced lung injury. (**A**) Schematic of experimental design. (**B**) Survival curve of adoptive Treg cell transfer experiments. *n* = 5 (young — PBS), 14 (aged — PBS), 9 (young into young), 9 (aged into aged), 9 (aged into young) and 11 (young into aged) animals per group. (**C**) Schematic of experimental design. (**D**) Survival curve of adoptive Treg cell transfer experiments in *Foxp3^DTR^* mice. *n* = 7 to 8 animals per group except for the *Foxp3^DTR^* group (*n* = 3). DTx denotes diphtheria toxin. Survival curves of mice compared using log-rank (Mantel-Cox) test. *P < 0.05; **P < 0.005.

We and others have identified an essential role for Treg cells in orchestrating resolution and repair of acute lung injury (9–13). Having established that aged mice failed to repair the injured lung, we next sought to determine whether this finding is due to age-related features altering the lung microenvironment or is driven by cell-autonomous, age-associated Treg cell factors. Thus, we performed heterochronic (age-mismatched) adoptive transfer of 1 × 10^6^ splenic young or aged Treg cells (~90% CD4^+^CD25^hi^Foxp3^+^, Supplemental Figure 3A) via retro-orbital injection into aged or young mice 24 hours after influenza infection (Figure 3A). Notably, adoptive transfer of young Treg cells into aged hosts resulted in improved survival compared with aged mice that received isochronic (age-aligned) adoptive transfer of aged Treg cells. Conversely, adoptive transfer of aged Treg cells into young hosts worsened their survival compared with isochronic adoptive transfer of young Treg cells (Figure 3B).

To further characterize the effect of age on the splenic Treg cells used for adoptive transfer, we performed flow cytometry characterization of their Treg cell phenotype. In the spleen and other tissues, Treg cells can be phenotypically subdivided into resting or central Treg (cTreg) cells, which comprise the majority of the Treg cell pool in lymphoid organs, and activated or effector Treg (eTreg) cells, which can migrate to nonlymphoid organs upon stimulation. Aged splenic Treg cells exhibited a significantly decreased percentage of cTreg cells and increased percentage of eTreg cells in both the naive and after influenza conditions (Supplemental Figure 3B). Additionally, to determine the rate of lung engraftment of splenic Treg cells after heterochronic adoptive transfer, we quantified the number of transferred splenic Treg cells in the lungs of recipient mice after influenza infection. Interestingly, we found a small but significant increase in the number of young Treg cells that engrafted into the lungs of aged mice compared with aged Treg cells that were recovered from the lungs of young recipients (Supplemental Figure 3C).

We next turned to an inducible Treg cell depletion system using *Foxp3^DTR^* mice in order to eliminate Treg cells from recipients and specifically determine the age-related effect of donor Treg cells on the susceptibility to influenza-induced lung injury (Figure 3C). Heterochronic adoptive transfer of aged Treg cells into Treg cell–depleted *Foxp3^DTR^* mice 5 days after infection resulted in increased mortality compared with isochronic adoptive transfer of young Treg cells (Figure 3D). Combined, our findings demonstrated that the loss of Treg cell prorepair function in aged hosts was dominated by intrinsic, age-related changes in Treg cells and not conferred extrinsically by the aging lung microenvironment.

### Aging results in the loss of prorepair transcriptional programs in Treg cells during recovery from influenza-induced lung injury.

**Figure 4:**
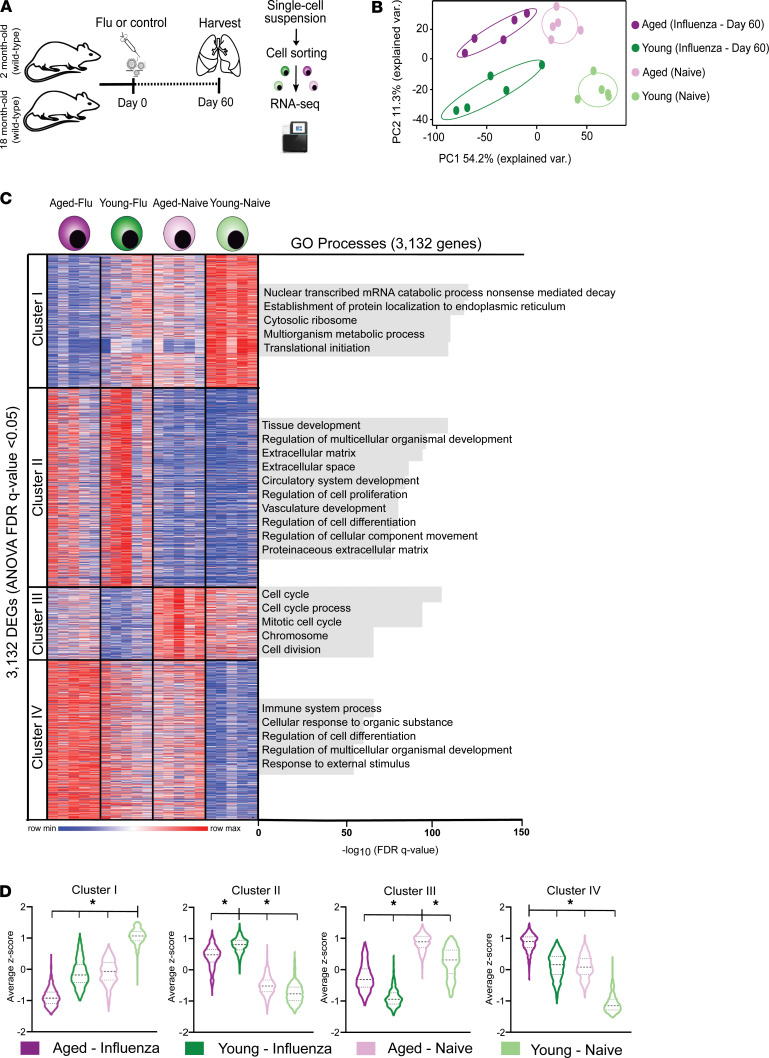
Young and aged Treg cells differ in their transcriptional response during recovery from influenza infection. (**A**) Schematic of experimental design. (**B**) Principal component analysis of 3,132 differentially expressed genes identified from a generalized linear model and ANOVA-like testing with FDR *q* value < 0.05. Ellipses represent normal contour lines with 1 standard deviation probability. (**C**) *K*-means clustering of 3,132 genes with an FDR *q* value < 0.05 comparing the cell populations from **B** with *k* = 4 and scaled as *z* scores across rows. Top 5 gene ontology (GO) processes derived from clusters I, III, and IV and top 10 GO processes derived from cluster II are annotated and ranked by –log_10_-transformed FDR *q* value. (**D**) Average *z* scores for the 4 clusters shown in **C**. Violin plots show median and quartiles. One-way ANOVA with 2-stage linear step-up procedure of Benjamini, Krieger, and Yekutieli with *Q* = 5%. *q < 0.05. *n* = 5 mice/group (young — naive, aged — naive, young — influenza, and aged — influenza) for all panels.

To further explore the mechanisms underpinning the age-related loss of Treg cell prorepair function after influenza infection, we performed gene expression profiling using RNA-Seq on flow cytometry–sorted lung CD3ε^+^CD4^+^CD25^hi^FR4^+^ Treg cells during the naive state or late recovery phase from influenza (day 60 after infection; Supplemental Figure 3D, Figure 4A, and ref. 23). We confirmed that sorted lung CD3ε^+^CD4^+^CD25^hi^FR4^+^ cells from young and aged hosts expressed high levels of canonical Treg cell signature genes (*Foxp3*, *Il2ra*, *Il2rb*, *Nrp1*, *Ikzf2*, and *Ctla4*) (Supplemental Figure 3E). Principal component analysis (PCA) of 3,132 differentially expressed genes after multiple-group testing with FDR *q* value less than 0.05 demonstrated tight clustering by group assignment; PC1 reflected the transcriptional response to influenza infection and PC2 reflected age (Figure 4B). *K*-means clustering of these differentially expressed genes demonstrated that cluster 2 was both the largest cluster and the one that defined the differential response to influenza infection between naive and influenza-treated mice (Figure 4C). Notably, genes from this cluster were significantly upregulated among young Treg cells compared with aged Treg cells after influenza infection (Figure 4D). Functional enrichment analysis revealed that this cluster was enriched for processes related to tissue and vasculature development and extracellular matrix formation (see Figure 4C, right), suggesting an enhanced reparative phenotype of young compared with aged Treg cells.

**Figure 5:**
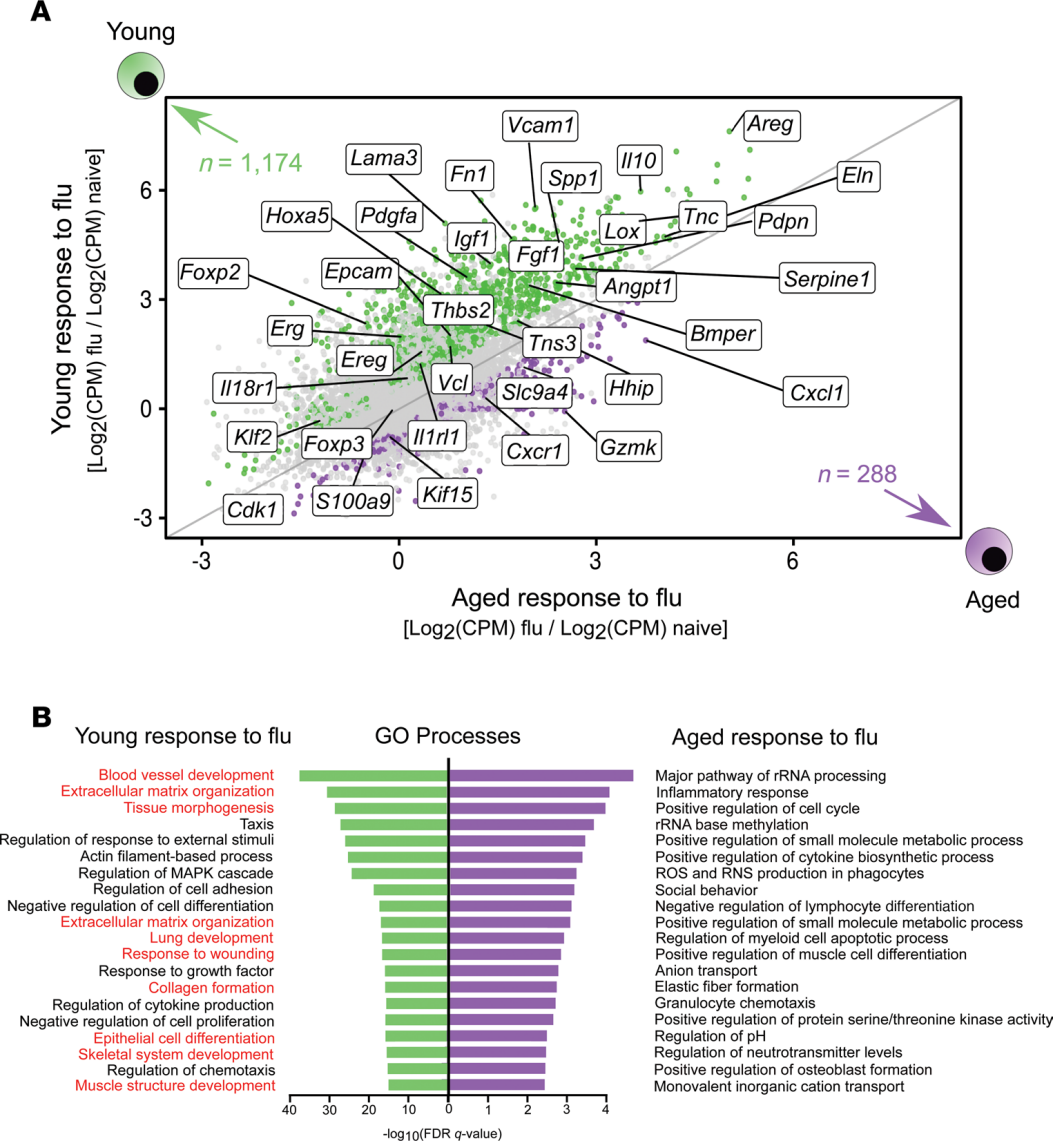
Young Treg cells upregulate a prorepair transcriptional program during recovery from influenza infection. (**A**) Fold change–fold change plot for the young Treg cell response to influenza infection versus the aged Treg cell response to influenza infection highlighting genes exhibiting *q* < 0.05 and fold change > 0.5 (green dots = young and purple dots = aged). Numbers of differentially expressed genes are indicated. (**B**) Top 20 gene ontology (GO) processes derived from differentially expressed genes (*q* < 0.05) from **A** for young Treg cells (1,174 genes) and aged Treg cells (288 genes) are annotated and ranked by –log_10_-transformed FDR *q* value. Red font denotes prorepair processes in young Treg cells *n* = 5 mice/group (young — naive, aged — naive, young — influenza, and aged — influenza) for all panels.

**Figure 6:**
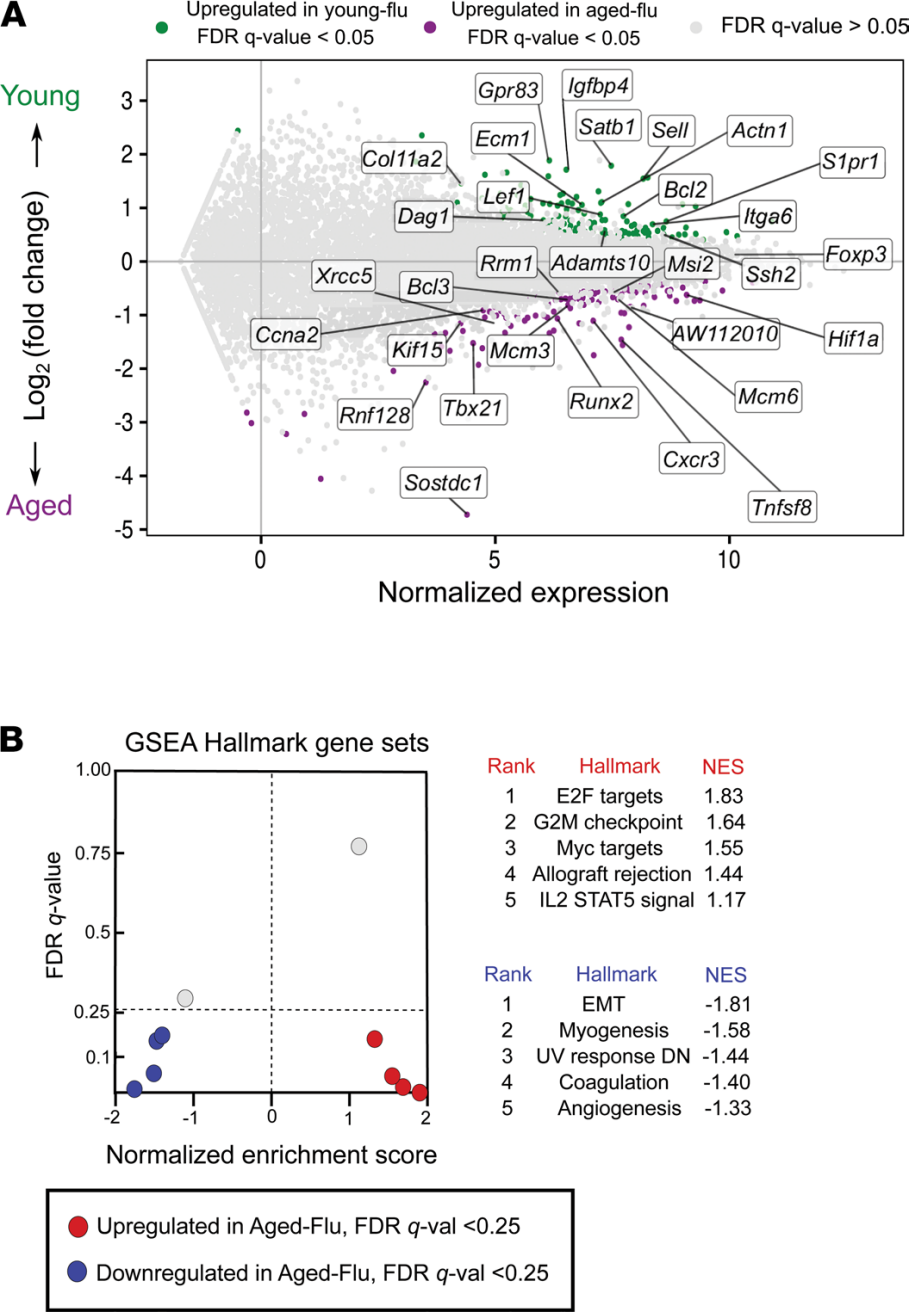
Aged Treg cells downregulate prorepair programs during recovery from influenza pneumonia when compared with young hosts. (**A**) MA plot comparing gene expression of young Treg versus aged Treg cells during the recovery phase from influenza infection. Genes of interest are annotated. (**B**) Gene set enrichment analysis (GSEA) dot plot highlighting key statistics (FDR *q* value and normalized enrichment score or NES) and gene set enrichment per phenotype. Genes were ordered by log_2_(fold change) and ranked by the aged Treg cell phenotype. Red dots denote gene sets with a positive enrichment score or enrichment at the top of the ranked list. Blue dots denote gene sets with a negative enrichment score or enrichment at the bottom of the ranked list. *n* = 5 mice/group (young — influenza and aged — influenza) for all panels.

We then performed an unsupervised analysis of the response to influenza infection from the naive state to recovery phase between young and aged Treg cells. This analysis revealed upregulation of 1,174 genes (log_2_[fold change] > 0.5, FDR *q* value < 0.05), mostly linked to lung development (including epithelial and endothelial cell differentiation), extracellular matrix organization, and wound healing (*Foxp2*, *Hhip*, *Klf2*, *Tns3*, *Hoxa5*, *Epcam*, *Erg*, *Bmper*, *Ereg*, *Lox*, *Tnc*, *Lama3*, and *Spp1*) in young hosts (Figure 5, A and B). We also found increased expression of genes associated with specialized Treg cell function in the maintenance of nonlymphoid tissue homeostasis and regenerative function (*Il1rl1* [encodes ST2, also known as IL33R], *Il18r1*, *Il10*, and *Areg*). Aged Treg cells demonstrated increased expression of cell cycle genes (*Kif15* and *Cdk1*), neutrophil chemotaxis (*Cxcr1*, *Cxcl1*, and *S100a9*), and cytotoxic effector function (*Gmzk*). Gene set enrichment analysis (GSEA) of the pairwise comparison between young and aged Treg cells during the recovery phase after influenza infection revealed that aged Treg cells downregulated repair-associated processes such as epithelial-mesenchymal transition, myogenesis, and angiogenesis compared with young hosts (Figure 6, A and B). This pairwise comparison demonstrated that although young Treg cells exhibited significantly increased expression of genes associated with naive (resting) state and lymphoid tissue markers akin to central Treg (cTreg) cell phenotype (*Lef1*, *Sell*, *Satb1*, *Bcl2*, *S1pr1*, *Gpr83*, and *Igfbp4*), aged Treg cells upregulated genes implicated in effector Th1, Th17, and T follicular regulatory differentiation (*Tbx21*/*Cxcr3*, *Hif-1a*, and *Sostdc1*, respectively); cell cycle (*Ccna2*, *Mmc3* and *Msi2*); T cell anergy (*Rnf128*); and DNA damage response (*Xrcc5* and *Rm1*) (Figure 6A). Collectively, these results revealed that aged Treg cells display a less robust reparative phenotype compared with young hosts and exhibit features of age-related maladaptive T cell responses, including effector T cell differentiation, cell cycle arrest, and DNA damage responses (24).

### Lung Treg cells from aged mice exhibit a less robust reparative response than lung Treg cells from young animals during recovery from influenza pneumonia.

**Figure 7:**
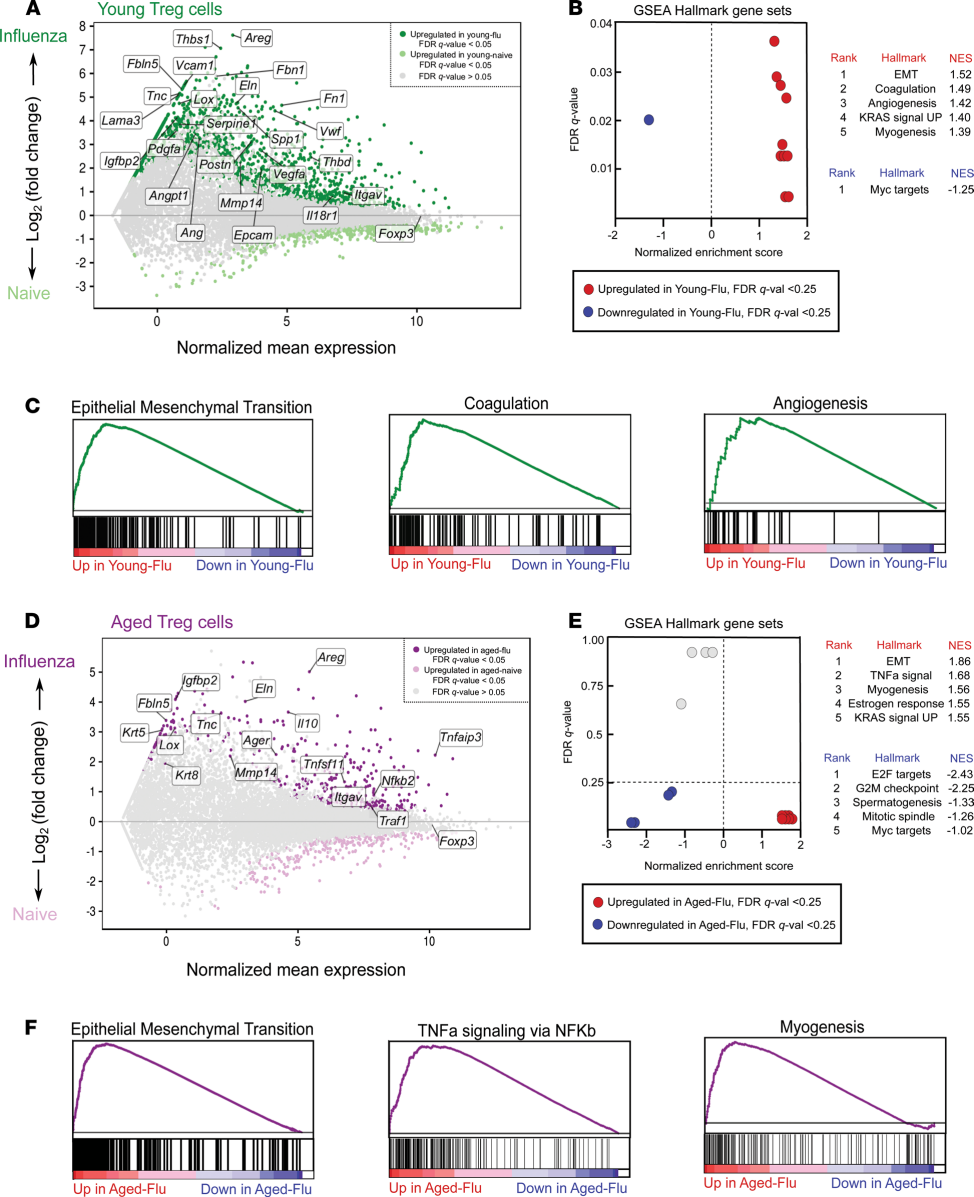
Aged lung Treg cells fail to upregulate a prorepair transcriptional program to the same extent as young Treg cells during recovery from influenza infection. (**A**) MA plot comparing gene expression of young Treg cells during the naive state with young Treg cells during the recovery phase of influenza infection. Genes of interest are annotated and *y* axis denotes fold-change dynamic range. (**B**) MA plot comparing gene expression of aged Treg cells during the naive state with aged Treg cells during the recovery phase of influenza infection. Genes of interest are annotated and *y* axis denotes fold-change dynamic range. Note that the *y* axis extends to +8 in **A** and +5 in **B**. (**C** and **D**) GSEA dot plot results highlighting key statistics (FDR *q* value and normalized enrichment score or NES) and enriched gene sets per phenotype. Genes were ordered by log_2_(fold change) and ranked by the young Treg cell–influenza (**C**) or aged Treg cell–influenza (**D**) phenotype. Red dots denote gene sets with a positive enrichment score or enrichment at the top of the ranked list. Blue dots denote gene sets with a negative enrichment score or enrichment at the bottom of the ranked list. (**E** and **F**) Top 3 GSEA positive enrichment plots for the young Treg cell–influenza (**E**) or aged Treg cell–influenza (**F**) phenotype. *n* = 5 mice/group (young — naive, aged — naive, young — influenza, and aged — influenza) for all panels.

**Figure 8:**
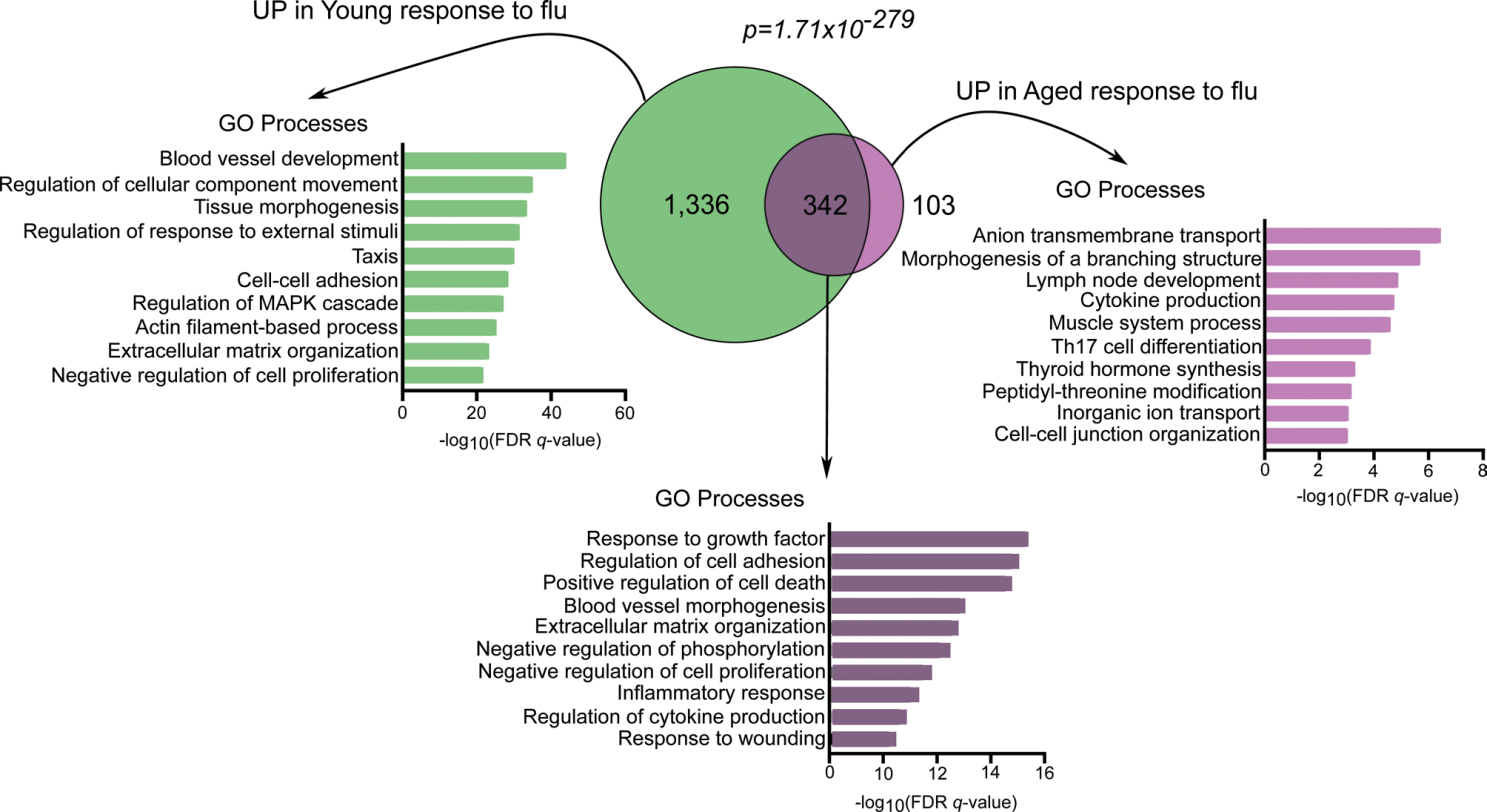
Comparison of enriched gene ontology processes during recovery from influenza pneumonia in young and aged Treg cells. Venn diagram partitioning into upregulated genes in young Treg cells during recovery from influenza infection (green, 1,336 genes), upregulated genes in aged Treg cells during recovery from influenza infection (light purple, 103 genes), and upregulated genes in both young and aged Treg cells during recovery from influenza infection (dark purple intersection, 342 genes). FDR *q* value < 0.05. A hypergeometric *P* value is shown. Top 10 gene ontology (GO) processes derived from genes in each partition of the Venn diagram are annotated and ranked by –log_10_-transformed FDR *q* value. *n* = 5 mice/group (young — influenza and aged — influenza).

To further investigate the age-related reparative function of Treg cells during the recovery phase of influenza infection, we performed pairwise comparisons of young and aged Treg cells from the naive state and recovery phase (day 60 after infection; Figure 7, A and D). We found that during the Treg cell response to influenza, there were 1,678 upregulated genes in young mice and only 445 upregulated genes in aged mice compared with their respective naive state (FDR *q* value < 0.05). GSEA revealed upregulation of prorepair hallmark processes in young and aged Treg cells during recovery from influenza infection (Figure 7, B, C, E, and F). We next compared the age-related transcriptional response to influenza infection and found 342 shared genes between both age groups that were associated with prorepair processes (Figure 8). The remaining 1,336 uniquely upregulated genes in young Treg cells were linked to reparative processes, in contrast with the 103 uniquely upregulated genes in aged Treg cells. Altogether, these results suggest that although aged Treg cells demonstrated a nominal reparative response during recovery from influenza infection, it was not as robust as the response exhibited by young Treg cells.

Previous studies have shown that Treg cells exhibiting a prorepair phenotype express high levels of the alarmin IL-33 receptor (ST2), proinflammatory cytokine receptor IL-18 (IL-18Rα or CD218α), and antiinflammatory cytokine IL-10 (13, 25). We measured the protein expression of these molecules by flow cytometry and found similar expression between young and aged Treg cells 60 days after influenza infection (Supplemental Figure 4A). We reasoned that measurement of only 3 markers linked to repair incompletely characterizes the complexity of the Treg reparative landscape during influenza-induced lung injury and recovery. Accordingly, we compared our young and aged Treg cell RNA-Seq dataset with previously published datasets describing reparative IL-10^+^ and IL-18Rα^+^ Treg cells (13). We found that reparative IL-10^+^ and IL-18Rα^+^ Treg cells were most similar to young Treg cells from our data set when compared with aged Treg cells (Supplemental Figure 4B). Indeed, the number of genes within the unique intersections of young and IL-10^+^ and IL-18Rα^+^ Treg cells was significantly higher than the number of genes within the unique intersections of aged and IL-10^+^ and IL-18Rα^+^ Treg cells. Genes within the unique intersections of young and IL-10^+^ and IL-18Rα^+^ Treg cells were enriched for gene ontology processes linked to reparative processes, including extracellular matrix remodeling, vascular remodeling, and tube morphogenesis (Supplemental Figure 4B). These results underscore our finding that the youthful Treg cell response during recovery from influenza pneumonia was defined by a more robust reparative phenotype compared with aged hosts.

### Treg cells from aged mice demonstrate a proinflammatory effector phenotype during recovery from influenza pneumonia.

**Figure 9:**
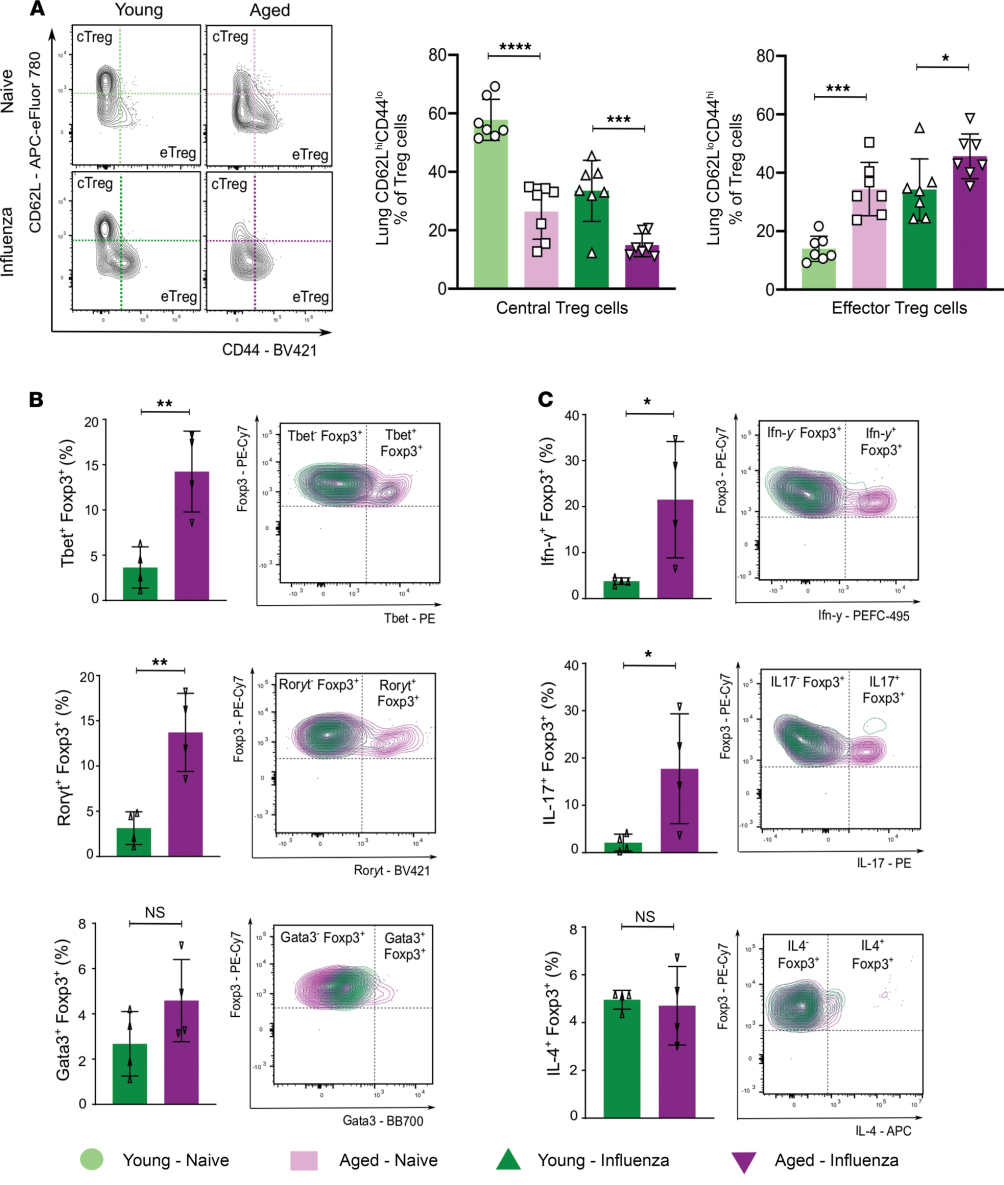
Aging results in induction of a Treg cell proinflammatory phenotype after recovery from influenza infection. (**A**) Representative flow cytometry contour plot analysis of central Treg (cTreg) cells and effector Treg (eTreg) cells. Percentage of lung cTreg cells (CD62L^hi^CD44^lo^) and lung eTreg cells (CD62L^lo^CD44^hi^) of lung Treg cells. *n* = 7 mice/group (young — naive, aged — naive, young — influenza, and aged — influenza). (**B**) Representative contour plots and pairwise comparison of percentage of CD4^+^Foxp3^+^ cells expressing T helper cell canonical transcription factors. (**C**) Representative contour plots and pairwise comparison of percentage of CD4^+^Foxp3^+^ cells expressing proinflammatory cytokines. Data presented as mean ± SD, 1-way ANOVA with Holm-Sidak post hoc testing for multiple comparisons (**A**) or Mann-Whitney test (**B** and **C**). *P < 0.05; **P < 0.005; ***P < 0.0005; ****P < 0.0001. NS, not significant. *n* = 4 mice/group (young — influenza and aged — influenza) for **B** and **C**.

Upon stimulation, Treg cells can exhibit phenotypic and functional adaptability through upregulation of transcription factors and chemokine receptors akin to effector T helper cell subsets (e.g., Th1, Th2, Th17, and T follicular regulatory; ref. 26). The resulting eTreg cells with Th-like phenotypes migrate to inflammatory nonlymphoid tissues and acquire transcriptional and functional programs that copy the T effector responses they intend to suppress (27, 28). Having observed transcriptional upregulation of effector-associated factors in the aged Treg cell response during recovery from influenza infection, we next performed flow cytometry analysis to measure canonical effector-associated transcription factors and cytokines at the protein level. During the recovery phase after influenza infection, aged mice exhibited a higher percentage of eTreg cells in their lungs compared with young hosts (Figure 9A). In the lung, compared with young CD4^+^Foxp3^+^ cells, aged cells exhibited significantly higher expression of the transcription factors Tbet and Ror-γt, canonical master regulators of Th1 and Th17 responses, respectively (Figure 9B). Surprisingly, intracellular cytokine profiling of these cells also demonstrated a significant increase in the percentage of Ifn-γ– and IL-17–producing CD4^+^Foxp3^+^ cells in the lungs of aged mice compared with young mice (Figure 9C).

Profiling of multiple well-established Treg cell suppressive markers, including *Foxp3*, showed no significant age-related difference during the recovery phase of influenza infection (Supplemental Figure 5A). In addition, aged and young Treg cells exhibited similar suppressive function in vitro, although there was a trend toward increased suppressive function in aged cells, as has been suggested by a previous report (Supplemental Figure 5B and ref. 29). These data suggest that Treg cells in the lungs of aged animals upregulate inflammatory effector programs during recovery from influenza. Although these effector programs are ostensibly protective during acute influenza-induced acute lung injury, they may represent an age-related maladaptive Treg cell response, leading to unremitting lung inflammation and injury during recovery.

### DNA methylation regulates the transcriptional prorepair response to influenza infection.

**Figure 10:**
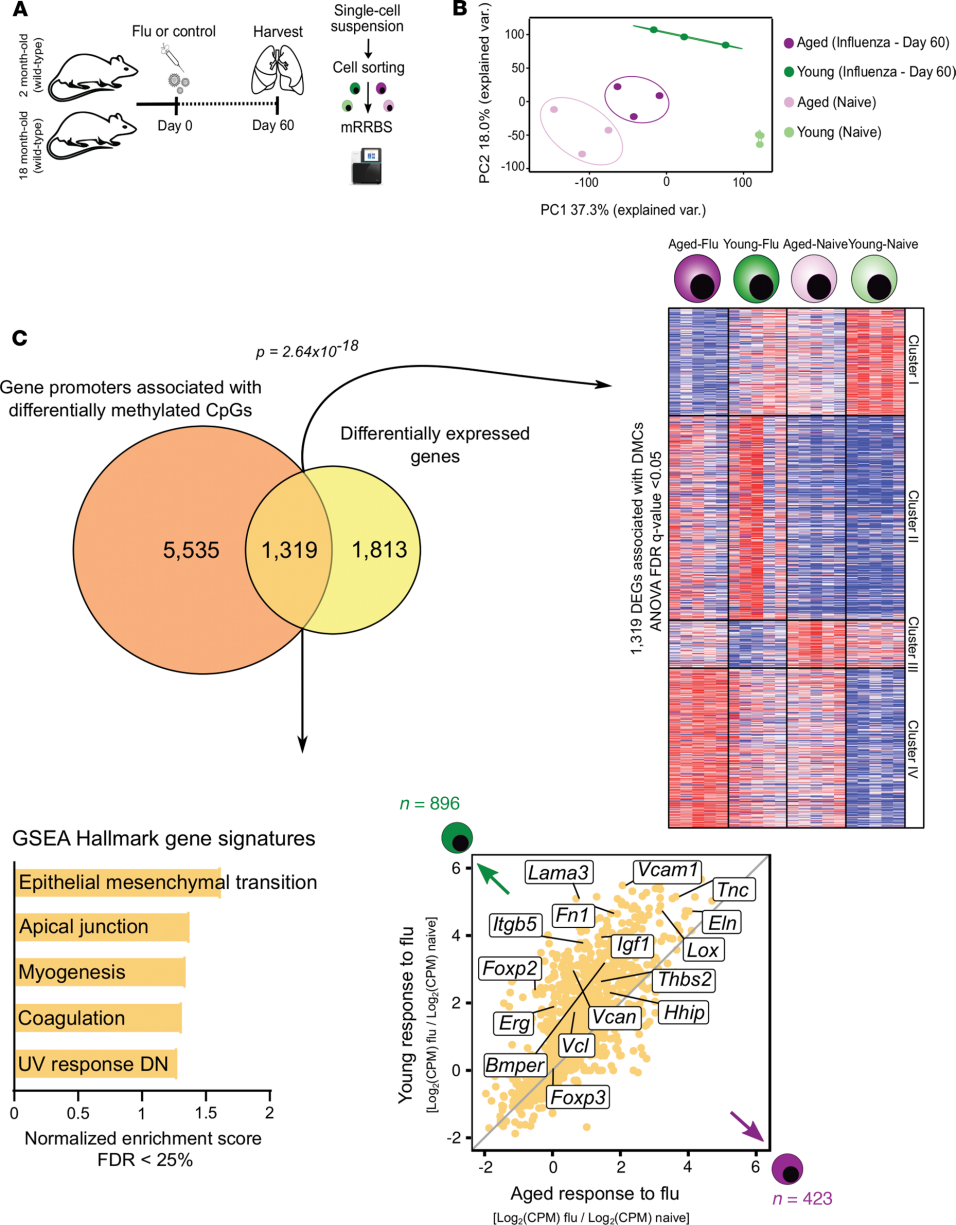
DNA methylation regulates the age-related prorepair gene signature during recovery from influenza infection. (**A**) Schematic of experimental design. (**B**) Principal component analysis of approximately 70,000 differentially methylated cytosines (DMCs) identified from a generalized linear model and ANOVA-like testing with FDR *q* value < 0.05. Ellipses represent normal contour lines with 1 standard deviation probability. *n* = 3 mice/group (young — naive, aged — naive, young — influenza, and aged — influenza). (**C**) Venn diagram partitioning into differentially expressed genes or DEGs (yellow, 1,813 genes), gene promoters containing DMCs (dark orange, 5,535 genes), and genes that are both DEGs and have gene promoters containing DMCs (light orange intersection, 1,319 genes). Promoters were defined as 1 kb surrounding the transcription start site. A hypergeometric *P* value is shown. *K*-means clustering of 1,319 genes with an FDR *q* value < 0.05. Fold change–fold change plot for young Treg cell response to influenza versus aged Treg cell response to influenza infection highlighting methylation-regulated DEGs. GSEA results showing the top 5 positively enriched gene sets with an FDR *q* value < 0.25. Genes were ordered by log_2_(fold change) and ranked by the young Treg cell phenotype. *n* = 5 mice/group (young — naive, aged — naive, young — influenza, and aged — influenza).

In addition to representing one of the hallmarks of aging, epigenetic phenomena such as DNA methylation regulate the development, differentiation, and functional specialization of T cell lineages, including Treg cells (14–16, 30). Therefore, we reasoned that age-related changes to the Treg cell DNA methylome could inform the divergent prorepair transcriptional response observed between young and aged Treg cells after influenza infection. We performed genome-wide 5′-cytosine–phosphate–guanine-3′ (CpG) methylation profiling with modified reduced representation bisulfite sequencing of sorted lung Treg cells during the naive state or recovery phase after influenza infection (day 60, Figure 10A). PCA of approximately 70,000 differentially methylated cytosines (FDR *q* value < 0.05) revealed tight clustering according to group assignment; the main variance across the data set (PC1) reflected methylation changes due to age (Figure 10B), consistent with prior studies (17, 31). We next identified genes that were both differentially expressed and contained differentially methylated cytosines within their gene promoters (ANOVA, FDR *q* value < 0.05), finding 1,319 genes meeting this strict parameter threshold (Figure 10C). *K*-means clustering of these genes’ expression levels revealed similarity to the overall heatmap of differentially expressed genes in Figure 4C, suggesting a strong correlation between differential DNA methylation and transcription. GSEA of these genes demonstrated that this methylation-regulated gene expression program was associated with prorecovery processes and was significantly skewed toward young Treg cells (Figure 10C). Combined, these results support the notion that age-related DNA methylation regulates the reparative transcriptional regulatory network during recovery from influenza-induced lung injury.

## Discussion

We sought to unambiguously address the paradigm of how aging affects Treg cell function during recovery from influenza pneumonia. We used heterochronic (age-mismatched) Treg cell adoptive transfer after influenza infection to establish that the age-related prorepair function of these cells is determined by cell-autonomous mechanisms. While adoptive transfer of young Treg cells into aged or Treg cell–depleted hosts demonstrated a salutary effect, the transfer of aged cells into young or Treg cell–depleted hosts had a detrimental impact on mortality. Comprehensive transcriptional and DNA methylation profiling revealed age-related epigenetic repression of the youthful reparative gene expression profile and activation of maladaptive responses in lung Treg cells among aged hosts.

The ongoing coronavirus disease 2019 (COVID-19) pandemic caused by SARS-CoV-2 represents an unprecedented challenge for the scientific community to identify novel pharmacotherapies and strategies for effective disease management. Most studies have addressed the early mechanistic events leading to viral pneumonia–induced lung injury, yet failed to develop efficacious therapies to improve outcomes among patients with viral ARDS. Thus, we focused our experimental design on the late stages of recovery from influenza infection with the goal of investigating potentially novel reparative pathways that could be leveraged to enhance lung resilience to viral pneumonia in older hosts. Both influenza A virus– and SARS-CoV-2–induced lung injury disproportionately affect the elderly, who comprise the greatest proportion of infection-related deaths (1, 3, 32, 33). Here, we found that similar to human epidemiological data and previous preclinical murine studies, aged mice exhibited increased susceptibility and impaired recovery after influenza infection (4, 19). Injury to alveolar epithelial type 1 and 2 and endothelial cells disrupts the tight gas-exchange barrier, causing accumulation of fluid and proinflammatory mediators in the alveolar space, a hallmark of ARDS pathophysiology (5). Notably, we found that during late recovery from influenza infection, aged hosts demonstrated a decreased number of AT2 cells and endothelial cells compared with young animals, suggesting that failure to repopulate the alveolar lining contributes to the observed age-related impairment in recovery. Severe influenza infection leads to a robust expansion of Krt5^+^ cells, which migrate distally to form cyst-like structures or pods intended to cover the damaged alveolar wall (22). These pods persist long after the initial infection, lack the capacity to generate a functional alveolar epithelium, and therefore constitute an insufficient reparative response to injury (22). Here, we found that aged animals displayed an increased percentage of Krt5^+^ cells during the recovery phase of influenza-induced lung injury, which reflects the dysregulated repair response among aged hosts.

Over the past decade, Tregs have emerged as key mediators of wound healing and tissue regeneration (7, 18, 34). This group of specialized cells has been primarily known for the ability to suppress effector immune cell subsets, leading to resolution of inflammation, but these cells are also capable of directly affecting tissue regeneration through production of prorepair mediators, such as amphiregulin and keratinocyte growth factor (13, 25, 35). Investigators have demonstrated that aging can negatively affect the composition and function of the Treg cell pool throughout the lifespan, rendering them inefficient as mediators of tissue repair (36). This decline might occur through cell-autonomous mechanisms that result in altered trafficking to the lung and T cell maladaptive responses that lead to increased susceptibility to disease. For instance, loss of stemness accompanied by differentiation into proinflammatory Th1/Th17 phenotypes, activation of DNA damage responses, and the senescence secretome are among some of the T cell maladaptations that result from the mounting challenges to which the T cell repertoire is exposed over a lifetime (24). These T cell maladaptive changes could also result from an age-related decline in stromal signals and circulating factors from the tissue microenvironment that either affect T cell function directly or render the microenvironment resistant to T cell responses. Our heterochronic adoptive Treg cell transfer experiments definitively address this paradigm, ascertaining that the observed age-related Treg cell dysfunction is due to cell-autonomous mechanisms and dominant over the aged pulmonary microenvironment. Our data demonstrated that aging not only imparted a loss of prorecovery Treg cell function, but also a gain of some of these maladaptive features compared with young hosts. Future studies should aim to address the respective contribution of loss of reparative function or gain of maladaptive function to the overall phenotype and function of aged Treg cells during virus-induced lung injury and recovery.

What are the molecular mechanisms underpinning the age-associated Treg cell loss of reparative function in the lung after influenza infection? Gene expression profiling of lung Treg cells during the recovery phase of influenza infection revealed that young Treg cells significantly upregulated genes (compared with aged Treg cells) linked to biological processes associated with a robust prorepair signature, including extracellular matrix organization, alveologenesis, and vasculogenesis. Here, we demonstrated that the young Treg cell prorepair program was dominated by *Areg* expression and other genes related to the above-mentioned reparative processes. Interestingly, we found no difference when comparing the suppressive phenotype and function of young versus aged Treg cells after influenza infection and during steady state. These results suggest that the reparative program of Treg cells is separable and distinct from their suppressive program, an important observation that informs the development of Treg cell–based immunotherapies to target molecular pathways regulating their reparative function. In regard to aged Treg cells, we found that although capable of upregulating a prorepair program after influenza infection, the response was far less robust than the youthful reparative response. Moreover, aged Treg cells displayed increased expression of genes associated with an effector phenotype with increased expression of canonical Th1 and Th17 lineage markers (Tbet/IFN-γ and Ror-γt/IL-17, respectively). Whether this finding represents an age-related functional adaptability of Treg cells after influenza infection or it is the result of Treg cell lineage instability leading to effector differentiation remains unknown.

For our studies, because of the practical limitations of aging *Foxp3* genetic reporter animals, we identified Treg cells as CD3ε^+^CD4^+^CD25^hi^FR4^+^, a strategy validated by our transcriptomics data (Supplemental Figure 3E) and previous work (23). Nevertheless, bulk RNA-Seq analysis averages gene expression profiles across a heterotypic Treg cell pool and presents them as a monotypic population, constituting a limitation to our data interpretation. Future studies should implement single-cell technologies to accurately describe the heterogeneity of the Treg cell landscape during virus-induced lung injury and recovery, which will allow investigators to identify Treg subpopulations that can be leveraged for cell-based therapies.

Establishment of a Treg cell–specific DNA methylation pattern at key genomic loci is necessary to maintain the lineage stability and immunosuppressive function of Treg cells (15, 30). Epigenomic profiling has revealed that Treg cell–specific alterations in methylation patterning modulate Treg cell transcriptional programs and increase susceptibility to human autoimmune diseases (37). Whether epigenetic phenomena have a similar regulatory role in modulating the Treg cell reparative gene expression program remains unknown. Here, we used an unsupervised bioinformatics analysis to uncover a Treg cell–specific methylation-regulated transcriptional program enriched for reparative processes during recovery from influenza infection. Our computational integrative approach provides inferential evidence that age-related DNA methylation can modify the expression of genes linked to prorepair processes in Treg cells but does not prove causality and therefore represents a limitation of our study. Future research could focus on leveraging epigenome editing technologies to establish the causality of age-related, Treg cell–specific DNA methylation patterns in controlling their regenerative function (38).

In conclusion, our study establishes that aging imparts cell-autonomous dysfunction to the reparative capacity of Treg cells after influenza pneumonia. The youthful reparative transcriptional response of Treg cells is dominated by processes linked to epithelial and endothelial cell repair and extracellular matrix remodeling and demonstrates regulation by DNA methylation. Aged Treg cells exhibited a less robust reparative program and displayed features of maladaptive T cell responses. These findings carry important implications for the development of small molecule– and Treg cell–based therapeutics that promote restoration of lung architecture and function after viral pneumonia in our increasingly older population.

## Methods

### Mice.

Young (2–4 months) and aged (18–22 months) C57BL/6 mice were obtained from The National Institute on Aging Aged Rodent Colony. *Foxp3^DTR^* mice were purchased from The Jackson Laboratory (Jax 016958). Animals received water ad libitum, were housed at a temperature range of 20°C to 23°C under 14-hour light/10-hour dark cycles, and received standard rodent chow.

### Administration of influenza A virus and lung histopathology.

WT C57BL/6 mice were anesthetized with isoflurane and intubated using a 20-gauge angiocatheter cut to a length that placed the tip of the catheter above the carina. Mice were instilled with a mouse-adapted influenza A virus (A/WSN/33 [H1N1]; 3 PFU/mouse or 2 PFU/mouse for *Foxp3^DTR^* mice, in 50 μL of sterile PBS), as previously described (39).

To prepare organ tissues for histopathology, the inferior vena cava was cut and the right ventricle was perfused in situ with 10 mL of sterile PBS. A 20-gauge angiocatheter was then sutured into the trachea via a tracheostomy. The lungs were removed en bloc and inflated to 15 cmH_2_O with 4% paraformaldehyde. Next, 5 μm sections from paraffin-embedded lungs were stained with H&E and examined using light microscopy with a high-throughput, automated slide imaging system, TissueGnostics (TissueGnostics GmbH).

### Tissue preparation, flow cytometry analysis, and sorting.

Single-cell suspensions from harvested mouse lungs were prepared and stained for flow cytometry analysis and flow cytometry sorting as previously described using the reagents listed in Supplemental Table 1 (see also refs. 15, 40, 41). The CD4^+^ T Cell Isolation Kit, mouse (Miltenyi Biotec) was used to enrich CD4^+^ T cells in single-cell suspensions prior to flow cytometry sorting. Cell counts of single-cell suspensions were obtained using a Cellometer with AO/PI staining (Nexcelom Bioscience) before preparation for flow cytometry. Data acquisition for analysis was performed using a BD Symphony A5 instrument with FACSDiva software (BD Biosciences). Cell sorting was performed using the 4-way purity setting on BD Biosciences FACSAria SORP instruments with FACSDiva software. Analysis was performed with FlowJo v10.6.1 software.

### Cytokine measurements.

Lungs were harvested from young and aged mice and a single-cell suspension was obtained. Red blood cells were removed with ACK Lysis Buffer (Thermo Fisher Scientific) following the manufacturer’s instructions. Single-cell suspensions were plated on 12-well cell culture plates (Thermo Fisher Scientific) at a concentration of 1 × 10^6^ cells/mL with RPMI plus 2 μL/mL Leukocyte Activation Cocktail with GolgiPlug (BD Biosciences) and incubated for 4 hours at 37°C. After incubation, cells were resuspended in PBS and stained with a viability dye and subsequently with fluorochrome-conjugated antibodies directed at IFN-γ (clone XMG1.2), IL-17 (clone TC11-18H1), IL-10 (clone JES5-16E3), and IL-4 (clone 11B11). Data acquisition and analysis were performed as described above.

### Treg cell isolation and adoptive transfer.

Splenic CD4^+^CD25^+^ Treg cells were isolated from euthanized young (2–4 months) and aged (18–22 months) C57BL/6 mice by use of magnetic separation with the EasySep Mouse CD4^+^CD25^+^ Regulatory T Cell Isolation Kit II (STEMCELL Technologies) according to the manufacturer’s instructions. A separate group of young and aged C57BL/6 mice were challenged with 3 PFU/mouse of influenza A virus as previously described (39). Twenty-four hours later, a single-cell suspension of isolated 1 × 10^6^ splenic Treg cells in 100 μL of sterile PBS was obtained and transferred via retro-orbital injection into the influenza-treated mice. *Foxp3^DTR^* mice were challenged with 2 PFU/mouse of influenza A virus. Diphtheria toxin (List Biologicals) was administered via i.p. injection in 100 μL of sterile PBS in the following doses and days relative to influenza A virus infection (day 0): 50 μg/kg on day –2 and 10 μg/kg every 48 hours starting on day 0 and ending on day 28 after infection. Five days later, 1 × 10^6^ young or aged splenic Treg cells in 100 μL of sterile PBS were transferred via retro-orbital injection into the influenza-treated *Foxp3^DTR^* mice.

### RNA-Seq.

Flow cytometry–sorted lung Treg cells were pelleted in RLT plus buffer with 1% 2-mercaptoethanol and stored at –80°C until RNA extraction was performed. The Qiagen AllPrep DNA/RNA Micro Kit was used for simultaneous isolation of RNA and DNA (15, 42). RNA quality was assessed using a 4200 TapeStation System (Agilent Technologies). mRNA was isolated from purified 1 ng total RNA using oligo-dT beads (New England Biolabs, Inc). The NEBNext Ultra RNA kit was used for full-length cDNA synthesis and library preparation. Libraries were pooled, denatured, and diluted, resulting in a 2.0 pM DNA solution. PhiX control was spiked in at 1%. Libraries were sequenced on an Illumina NextSeq 500 instrument using the NextSeq 500 High Output reagent kit (1 × 75 cycles). For RNA-Seq analysis, FASTQ reads were demultiplexed with bcl2fastq v2.17.1.14, trimmed with Trimmomatic v0.38 (to remove low-quality basecalls), and aligned to the *Mus musculus* or mm10 (GRCm38) reference genome using TopHat v.2.1.0. Resultant bam files were imported into SeqMonk v1.45.4 to generate raw count tables with the RNA-Seq quantitation pipeline and filtered by protein-coding genes. Annotated probe reports from SeqMonk were imported into RStudio for downstream analysis. Differential gene expression analysis was performed with the edgeR v3.28.1 R/Bioconductor package using R v3.6.3 and RStudio v1.2.1578 (43). A genomic dataset visualization tool, Morpheus web interface (https://software.broadinstitute.org/morpheus/), was used to perform *K*-means clustering and heatmaps. Gene ontology analysis was performed by using either the Molecular Signatures Database (MSigDB) from the Broad Institute or the Metascape interface. GSEA was performed using the Broad Institute’s GSEA v4.0.3 software with the GSEAPreranked tool. A ranked gene list from young and aged phenotypes was ordered by log_2_(fold change) in average expression using 1,000 permutations and the Hallmark Gene Set database (44).

### Modified reduced representation bisulfite sequencing.

Genomic DNA was isolated from sorted lung Treg cells using a Qiagen AllPrep DNA/RNA Micro Kit. Endonuclease digestion, fragment size selection, bisulfite conversion, and library preparation were performed as previously described (15, 43, 45–47). Sequencing was performed on a NextSeq 500 instrument (Illumina). DNA methylation analysis and quantification were performed using Trim Galore! v0.4.3, Bismark v0.16.3, DSS v2.30.1 R/Bioconductor package and the bisulfite feature methylation pipeline from the SeqMonk platform. *Mus musculus* or mm10 (GRCm38) was used as the reference genome.

### Lymphocyte suppression assay.

In vitro Treg cell suppression assays were performed as previously described (9, 48). In brief, splenic CD4^+^CD25^+^ cells (~90% Foxp3^+^) were isolated using the EasySep Mouse CD4^+^CD25^+^ Regulatory T Cell Isolation Kit II (STEMCELL Technologies), incubated with a 1:1 ratio of MACSiBeads (Miltenyi Biotec) coated with anti-CD3/anti-CD28 and cocultured with differing ratios of Treg to CD4^+^ T effector (Teff) cells labeled with CellTrace Violet (Thermo Fisher Scientific), which were obtained from 8-week-old WT mice. After 48 hours, effector T cell proliferation was assayed by flow cytometry analysis.

### Viral plaque assay.

Viral plaque assays were performed as we previously described (49). In brief, MDCK cells were cultured in 6-well plates and grown to 100% confluence. Lung homogenates were obtained from mice at 14 days after infection. MDCK cells were incubated with serial dilutions of lung homogenates or control influenza A virus in DMEM plus 1% BSA at 37°C for 2 hours. Replacement media (4 mL/well) composed of DMEM, autoclaved Avicel 2.4%, and *N*-acetyl-trypsin was added and incubated for 3 days. The media were subsequently removed and plaques were visualized with blue-black staining (0.1% naphthalene black, 6% glacial acetic acid, and 13.6% anhydrous sodium acetate; ref. 49).

### Statistics.

All statistical tests are detailed either in Results or figure legends. Statistical analysis was performed using either GraphPad Prism v8.3.0 or R v3.6.3. Computational analysis was performed using Genomics Nodes and Analytics Nodes on Quest, Northwestern University’s High-Performance Computing Cluster. A *P* or *q* value of less than 0.05 was considered significant except for GSEA, in which 0.25 was considered significant.

### Data and material availability.

The raw and processed next-generation sequencing data sets have been uploaded to NCBI’s Gene Expression Omnibus (GEO GSE151543). Code used for analysis of Supplemental Figure 4B is available at https://github.com/NUPulmonary/2020_MoralesNebreda

### Study approval.

All animal experiments and procedures were conducted in accordance with the standards established by the US Animal Welfare Act set forth in NIH guidelines and were approved by the IACUC at Northwestern University under protocols IS00006605 and IS00012955.

## Author contributions

LMN and BDS contributed to the conception, hypothesis delineation, and design of the study. LMN, KAH, MATA, NSM, JYSH, AMJ, RPA, HAV, YP, and BDS performed experiments/data acquisition and analysis. LMN and BDS wrote the manuscript.

## Supplementary Material

Supplemental data

## Version 1 02/18/2021

In-Press Preview

## Version 2 03/22/2021

Electronic publication

## Version 3 03/23/2021

Figure 5A was corrected.
