# Supplementary material for: Aging imparts cell-autonomous dysfunction to regulatory T cells during recovery from influenza pneumonia
Source: JCI Insight. 2021 Mar 22;6(6):e141690. doi: 10.1172/jci.insight.141690 (PMC8026188; doi:10.1172/jci.insight.141690)
Supplement: Supplemental data [file jciinsight-6-141690-s205.pdf]

Supplemental Data

Aging imparts cell-autonomous dysfunction to regulatory T cells during recovery from influenza pneumonia

Luisa Morales-Nebreda[1], Kathryn A. Helmin[1], Manuel A. Torres Acosta[1], Nikolay S. Markov[1], Jennifer Yuan-Shih Hu[1], Anthony M. Joudi[1], Raul Piseaux-Aillon[1], Hiam Abdala-Valencia[1], Yuliya Politanska[1], Benjamin D. Singer[1,2,3,*]

Author affiliations:
[1]Department of Medicine, Division of Pulmonary and Critical Care Medicine
Northwestern University Feinberg School of Medicine
Chicago, IL, 60611, USA

[2]Department of Biochemistry and Molecular Genetics
Northwestern University Feinberg School of Medicine
Chicago, IL, 60611, USA

[3]Simpson Querrey Institute for Epigenetics
Northwestern University Feinberg School of Medicine
Chicago, IL, 60611, USA

*To whom correspondence should be addressed:
Benjamin D. Singer, MD
Division of Pulmonary and Critical Care Medicine, Department of Medicine
Department of Biochemistry and Molecular Genetics
Simpson Querrey Institute for Epigenetics
Northwestern University Feinberg School of Medicine
Simpson Querrey, 5th Floor
Chicago, IL 60611 USA
benjamin-singer@northwestern.edu
Tel: (312) 503-4494
Fax: (312) 503-0411

Competing Interest Statement/Declaration of Interests

BDS has a pending patent application – US Patent App. 15/542,380, "Compositions and Methods to Accelerate Resolution of Acute Lung Inflammation." The other authors declare no competing interests.

Supplemental Figures

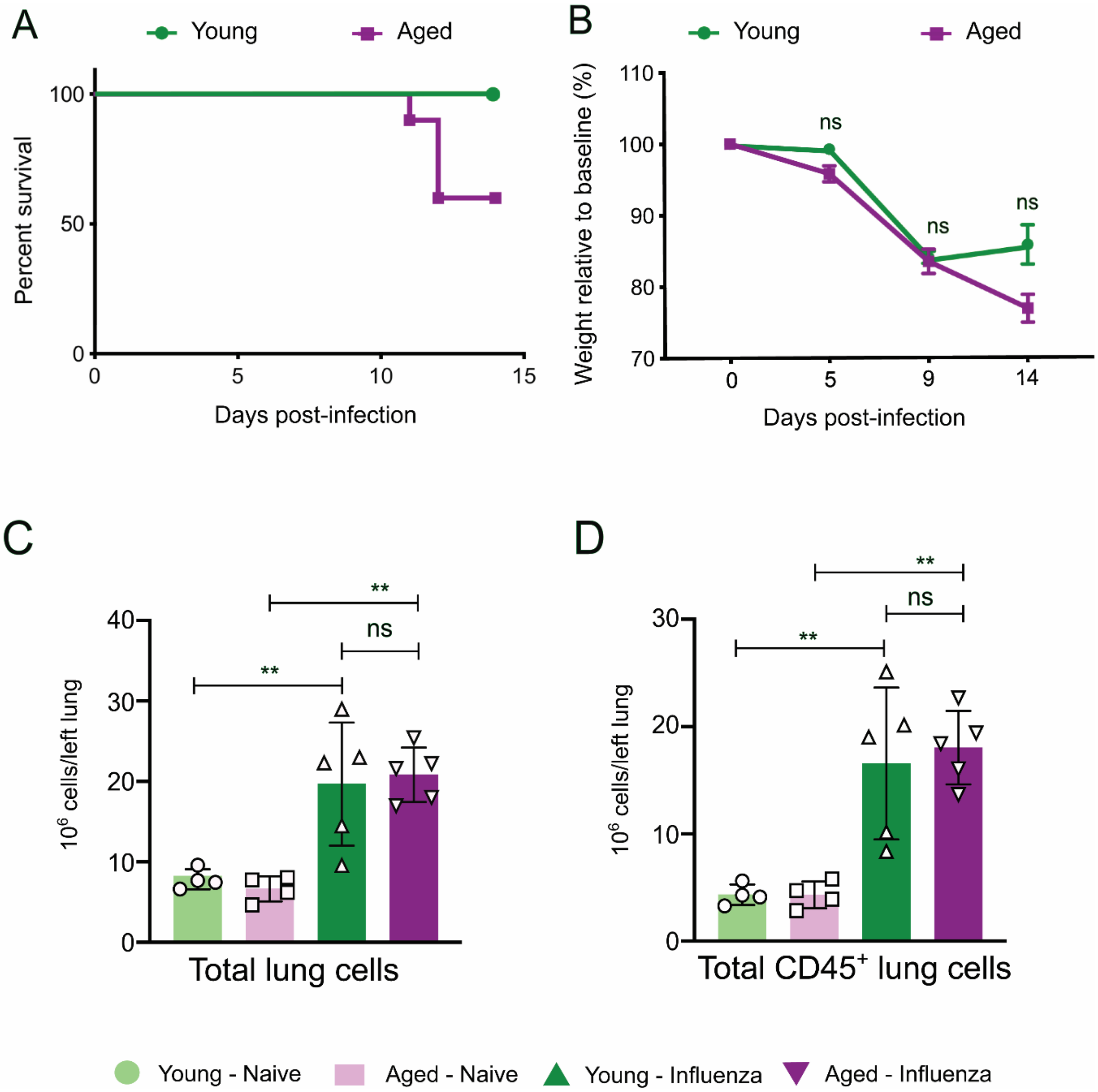
A
Young
Aged
Percent survival
100
50
0
0
5
10
15
Days post-infection
B
Young
Aged
Weight relative to baseline (%)
110
100
90
80
70
ns
ns
ns
0
5
9
14
Days post-infection
C
40
30
20
10
0
10^6 cells/left lung
**
ns
**
Total lung cells
D
30
20
10
0
10^6 cells/left lung
**
ns
**
Total CD45+ lung cells
Young - Naive
Aged - Naive
Young - Influenza
Aged - Influenza

Supplemental Figure 1. Aged and young mice demonstrate similar lung inflammation during early influenza-induced lung injury. (A) Survival curve of young (2-4 months, n = 10) and aged (18-22 months, n = 10) wild-type mice compared using the log-rank (Mantel-Cox) test. (B) Weight loss percentage from baseline in young (2-4 months, n = 10) and aged (18-22 months, n = 10) mice compared using a mixed-effects model (REML) with Sidak's *post-hoc* multiple comparisons test. Data presented as mean $\pm$ SEM. ns = not significant. (C) Flow cytometry quantitative analysis of total number of cells and (D) Total number of $CD45^+$ cells from left lung during the naïve state and early acute injury from influenza infection. Data presented as mean $\pm$ SD, one-way ANOVA with Holm-Sidak's *post-hoc* testing for multiple comparisons (C and D). ** $p < 0.005$, ns = not significant. n = 4-5 mice/group (young – naïve, aged – naïve, young – influenza and aged – influenza) for figures C and D.

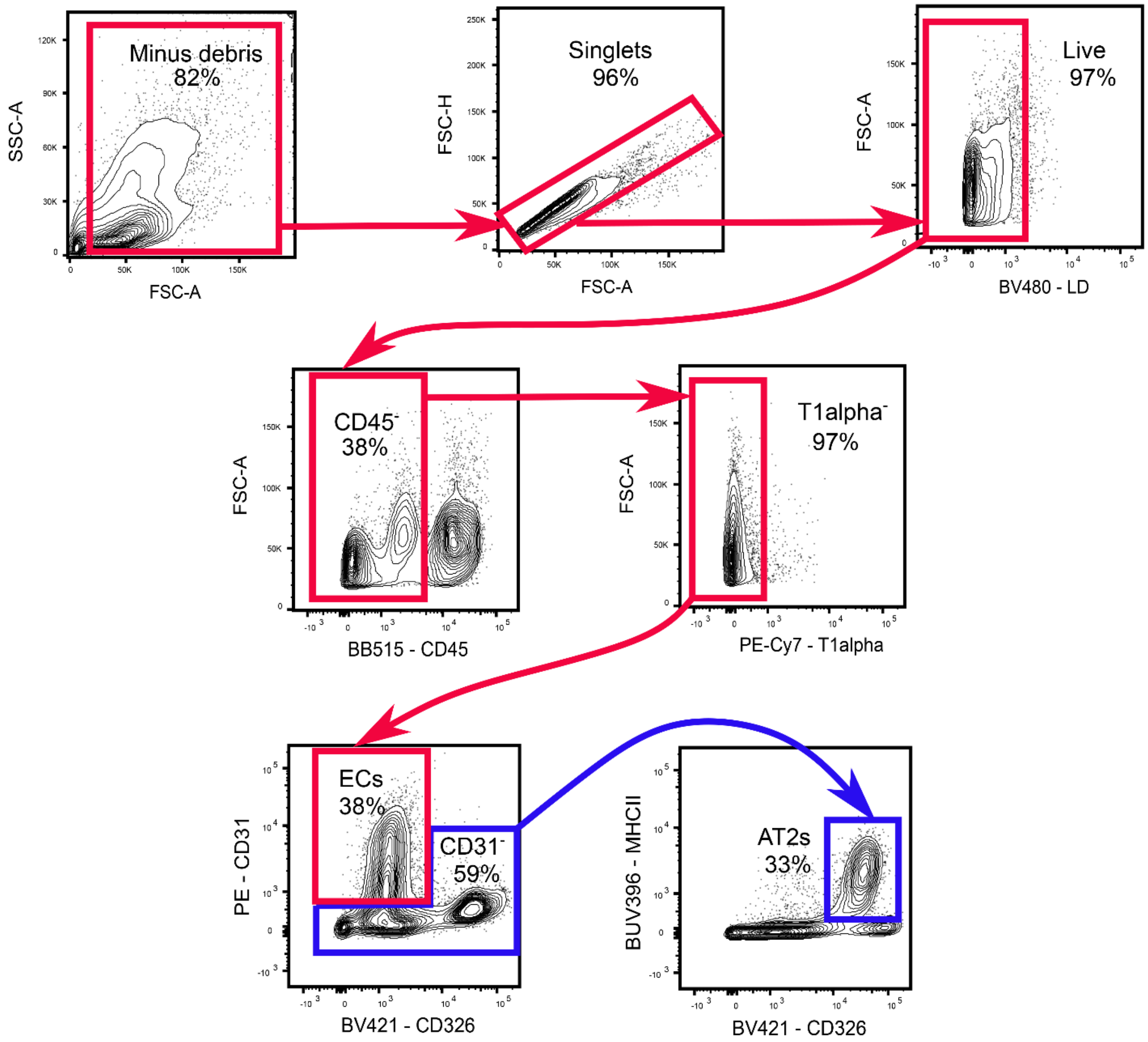
Minus debris
82%
SSC-A
FSC-A
Singlets
96%
FSC-H
FSC-A
Live
97%
FSC-A
BV480 - LD
CD45-
38%
FSC-A
BB515 - CD45
T1alpha-
97%
FSC-A
PE-Cy7 - T1alpha
ECs
38%
CD31-
59%
PE - CD31
BV421 - CD326
AT2s
33%
BUV396 - MHCII
BV421 - CD326

Supplemental Figure 2. Sequential gating strategy used for flow cytometry analysis of type II alveolar epithelial (AT2s) cells and endothelial cells (ECs).

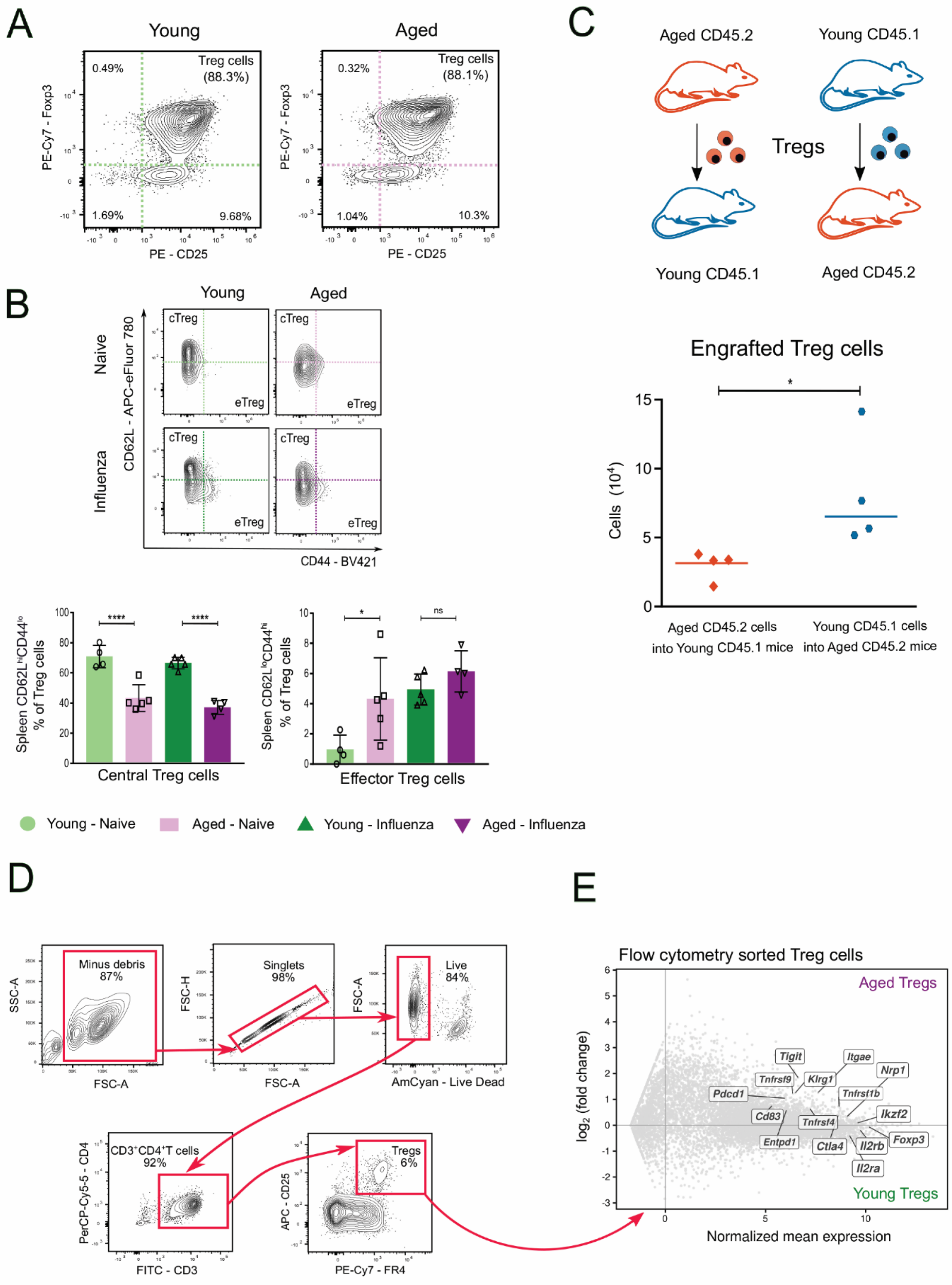
A
Young
Aged
Treg cells (88.3%)
Treg cells (88.1%)
0.49%
0.32%
1.69%
9.68%
1.04%
10.3%
PE-Cy7 - Foxp3
PE - CD25
B
Young
Aged
Naive
Influenza
CD62L - APC-eFluor 780
CD44 - BV421
cTreg
eTreg
Spleen CD62L^hiCD44^lo % of Treg cells
Central Treg cells
Spleen CD62L^loCD44^hi % of Treg cells
Effector Treg cells
****
*
ns
Young - Naive
Aged - Naive
Young - Influenza
Aged - Influenza
C
Aged CD45.2
Young CD45.1
Tregs
Young CD45.1
Aged CD45.2
Engrafted Treg cells
Cells (10^4)
Aged CD45.2 cells into Young CD45.1 mice
Young CD45.1 cells into Aged CD45.2 mice
D
Minus debris 87%
Singlets 98%
Live 84%
SSC-A
FSC-A
FSC-H
AmCyan - Live Dead
CD3+CD4+T cells 92%
Tregs 6%
PerCP-Cy5-5 - CD4
FITC - CD3
APC - CD25
PE-Cy7 - FR4
E
Flow cytometry sorted Treg cells
Aged Tregs
Young Tregs
log2 (fold change)
Normalized mean expression
Tigit
Itgae
Nrp1
Tnfrsf9
Klrg1
Pdcd1
Tnfrsf1b
Cd83
Tnfrsf4
Ikzf2
Entpd1
Ctla4
Il2rb
Foxp3
Il2ra

Supplemental Figure 3. Phenotypic characterization of the splenic Treg cell compartment and post-adoptive transfer engraftment efficiency. (A) Representative flow cytometry contour plot analysis of young and aged splenic Treg cells following magnetic bead isolation but prior to adoptive cell transfer. Treg cells ($CD25^{hi}Foxp3^{+}$) were gated on single, live and $CD4^{+}$ cells. (B) Representative flow cytometry contour plot phenotypic analysis of splenic central Treg (cTreg) cells and splenic effector Treg (eTreg) cells. Percentage of cTreg cells ($CD62L^{hi}CD44^{lo}$) and eTreg cells ($CD62L^{lo}CD44^{hi}$) of splenic Treg cells. *n* = 4 mice/group (young – naïve, aged – naïve, young – influenza and aged – influenza). (C) Quantification of engrafted splenic Treg cells in lung tissue 35 days post-influenza infection. *n* = 4 mice/group (aged CD45.2 into young CD45.1 and young CD45.1 into aged CD45.2). (D) Sequential gating strategy used for flow cytometry sorting of lung Treg cells following pre-sort enrichment for $CD4^{+}$ T cells via magnetic bead separation. (E) MA plot demonstrating the gene expression profile of young and aged lung Treg cells. Canonical Treg cell signature genes of interest are annotated. *n* = 5 mice/group (young – influenza and aged – influenza). Data presented as mean ± SD, one-way ANOVA with Holm-Sidak's *post-hoc* testing for multiple comparisons (B) or Mann-Whitney test (C). **** $p < 0.0001$, * $p < 0.05$, ns = not significant

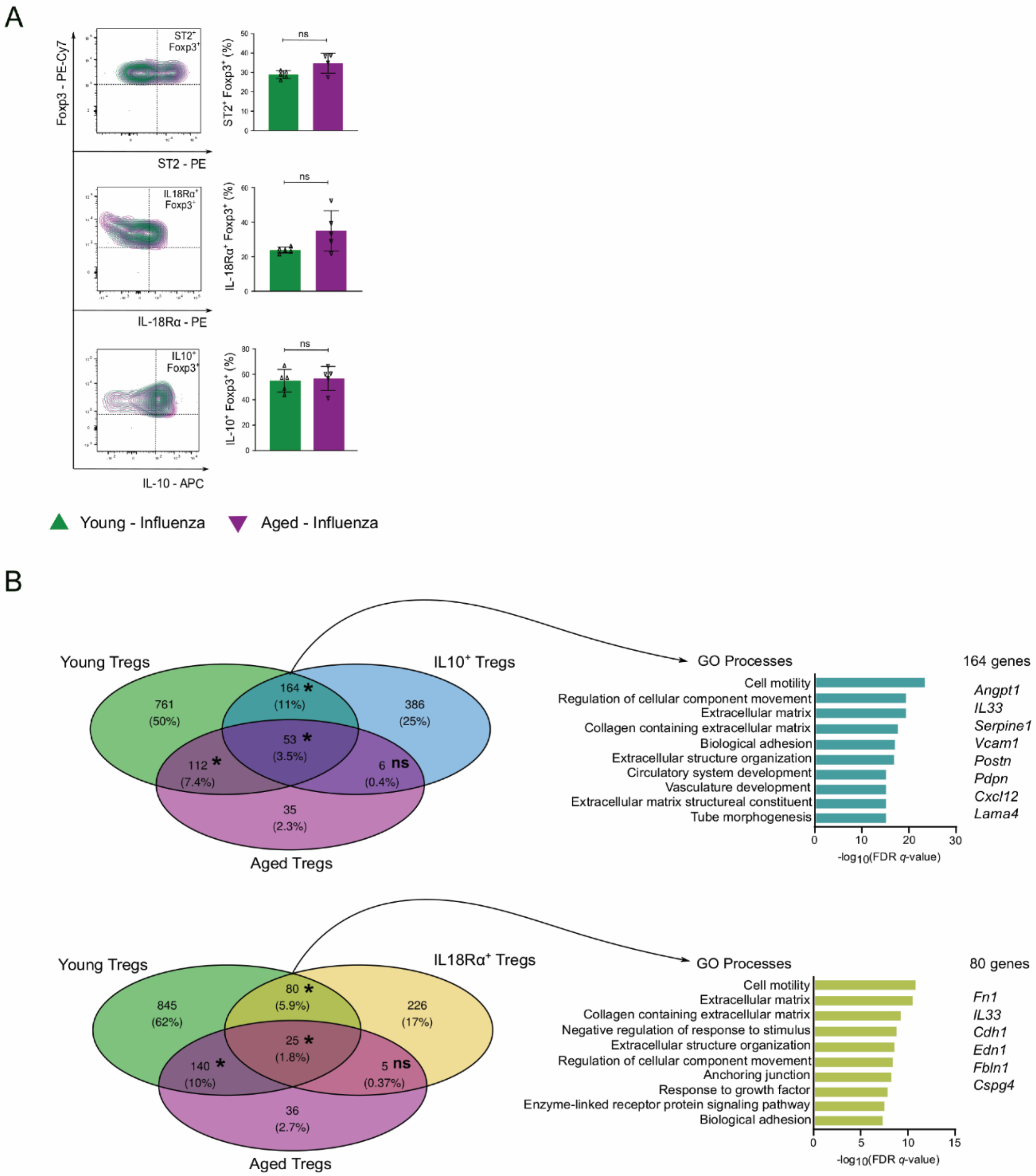
A
Foxp3 - PE-Cy7
ST2 - PE
ST2+ Foxp3+
ST2+ Foxp3+ (%)
ns
IL-18Rα - PE
IL18Rα+ Foxp3+
IL-18Rα+ Foxp3+ (%)
IL-10 - APC
IL10+ Foxp3+
IL-10+ Foxp3+ (%)
Young - Influenza
Aged - Influenza
B
Young Tregs
IL10+ Tregs
Aged Tregs
761 (50%)
164 * (11%)
386 (25%)
53 * (3.5%)
112 * (7.4%)
6 ns (0.4%)
35 (2.3%)
GO Processes
164 genes
Cell motility
Regulation of cellular component movement
Extracellular matrix
Collagen containing extracellular matrix
Biological adhesion
Extracellular structure organization
Circulatory system development
Vasculature development
Extracellular matrix structureal constituent
Tube morphogenesis
Angpt1
IL33
Serpine1
Vcam1
Postn
Pdpn
Cxcl12
Lama4
-log10(FDR q-value)
Young Tregs
IL18Rα+ Tregs
Aged Tregs
845 (62%)
80 * (5.9%)
226 (17%)
25 * (1.8%)
140 * (10%)
5 ns (0.37%)
36 (2.7%)
GO Processes
80 genes
Cell motility
Extracellular matrix
Collagen containing extracellular matrix
Negative regulation of response to stimulus
Extracellular structure organization
Regulation of cellular component movement
Anchoring junction
Response to growth factor
Enzyme-linked receptor protein signaling pathway
Biological adhesion
Fn1
IL33
Cdh1
Edn1
Fbln1
Cspg4
-log10(FDR q-value)

Supplemental Figure 4. Young Treg cells from the lung share a greater commonality with reparative Treg cell subsets than aged Treg cells. (A) Representative contour plots and pairwise comparison of percentage (%) of $CD4^+Foxp3^+$ cells expressing ST2 (IL33R), IL18Rα or IL10. Data presented as mean ± SD, Mann Whitney test. *n* = 4 mice/group (young – influenza and aged – influenza). (B) Venn diagram partitioning into upregulated genes in young Treg cells (761 genes), aged Treg cells (35 genes), $IL10^+$ Treg cells (386 genes), young and aged Treg cells (112 genes), young and $IL10^+$ Treg cells (164 genes), aged and $IL10^+$ Treg cells (6 genes) and young, aged and $IL10^+$ Treg cells (53 genes) (*Top panel*). Venn diagram partitioning into upregulated genes in young Treg cells (845 genes), aged Treg cells (36 genes), $IL18R\alpha^+$ Treg cells (226 genes), young and aged Treg cells (140 genes), young and $IL18R\alpha^+$ Treg cells (80 genes), aged and $IL18R\alpha^+$ Treg cells (5 genes) and young, aged and $IL18R\alpha^+$ Treg cells (25 genes) (*Bottom panel*). FDR q-value < 0.05 and fold-change (FC) > 2. Top 10 gene ontology (GO) processes derived from genes in young and $IL10^+$ Treg partition (*Top panel*), and young and $IL18R\alpha^+$ Treg partition (*Bottom panel*) of the Venn diagram are annotated and ranked by $-\log_{10}$-transformed FDR *q*-value. * $p < 0.05$, ns = not significant. *n* = 5 mice/group (young – influenza, aged – influenza, $IL10^+$ - influenza and $IL18R\alpha^+$ - influenza).

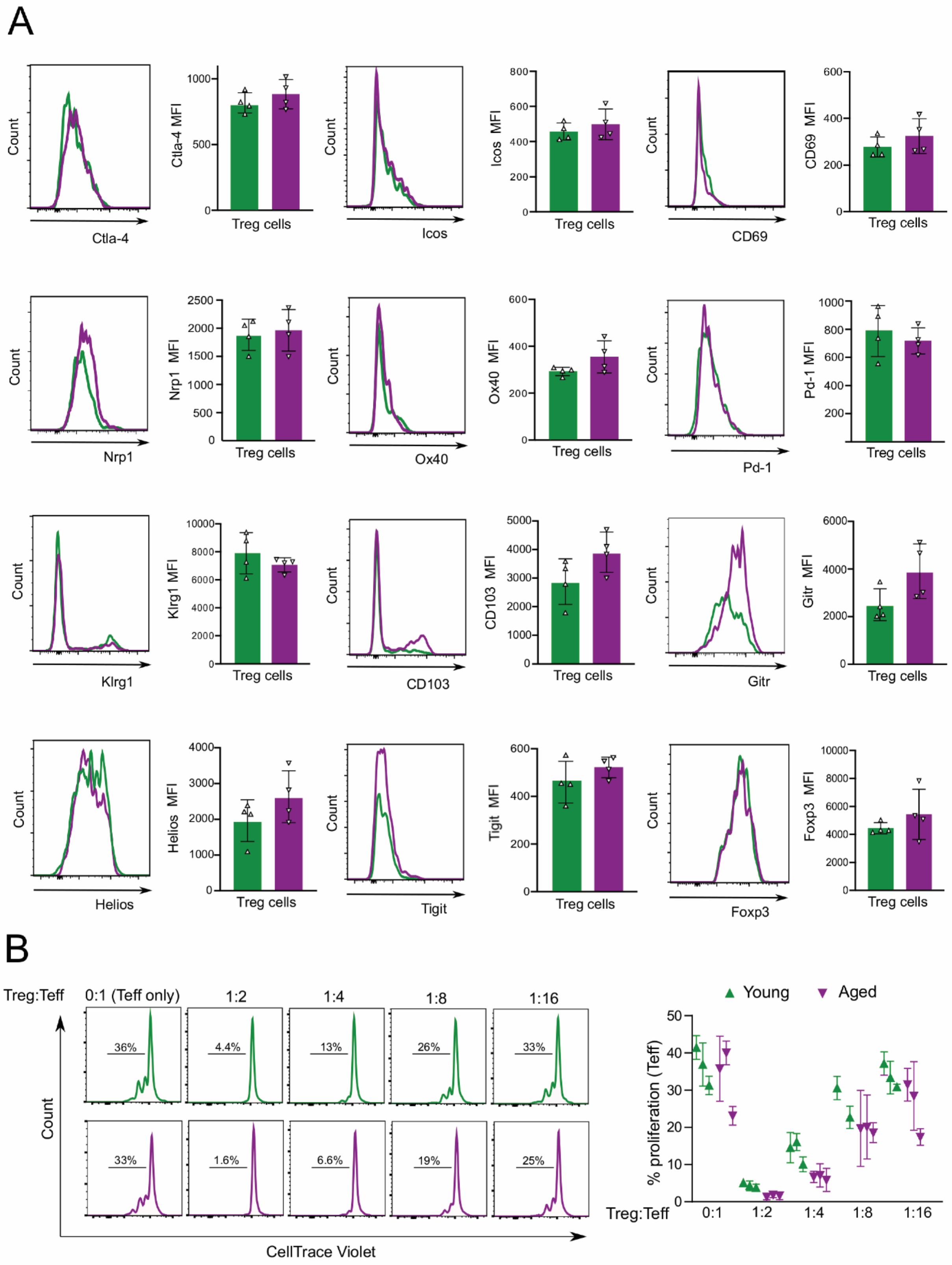
A
Count
Ctla-4
Ctla-4 MFI
Treg cells
Icos
Icos MFI
CD69
CD69 MFI
Nrp1
Nrp1 MFI
Ox40
Ox40 MFI
Pd-1
Pd-1 MFI
Klrg1
Klrg1 MFI
CD103
CD103 MFI
Gitr
Gitr MFI
Helios
Helios MFI
Tigit
Tigit MFI
Foxp3
Foxp3 MFI
B
Treg:Teff
0:1 (Teff only)
1:2
1:4
1:8
1:16
36%
4.4%
13%
26%
33%
33%
1.6%
6.6%
19%
25%
CellTrace Violet
Young
Aged
% proliferation (Teff)

Supplemental Figure 5. Aging does not result in changes in the suppressive phenotype and capacity of Treg cells. (A) Representative flow cytometry histogram plots of Treg cell-associated activation and suppressive markers in lungs of young and aged mice analyzed at day 60 post-influenza infection. *n* = 4 mice/group (young – influenza, aged – influenza). (B) CellTrace violet (CTV) dilution and percent (%) proliferation in $CD4^+$ T effector cells (Teff) co-cultured for 48 hours with varying ratios of Treg cells from young and aged animals. Data presented as mean ± SD (B). *n* = 3 mice per group in technical triplicates except for technical duplicates for the 1:8 young condition Neither the pairwise comparisons of each marker's median fluorescence intensity (MFI) in (A) nor the intra-dilutional comparisons in (B) were statistically significant by the Mann-Whitney test.

Supplemental Tables

Antigen/Reagent	Conjugate	Clone	Company	Catalog number
CD25	PE	PC61.5	eBioscience	12-0251-82
CD25	APC	PC61.5	eBioscience	17-0251-82
Foxp3	PE-Cyanine 7	FJK-16s	eBioscience	25-5773-82
CD3ε	APC	17A2	eBioscience	17-0032-82
CD3ε	FITC	145-2C11	eBioscience	11-0031-82
CD62L	APC-eFluor 780	MEL-14	eBioscience	47-0621-82
CD4	BUV395	GK1.5	BD	563790
CD4	PerCP-Cyanine 5.5	GK1.5	Biolegend	100433
CD4	APC-e780	GK1.5	Biolegend	100411
CD44	BUV373	IM7	BD	612779
CD44	BV421	IM7	Biolegend	103039
Tigit	PerCP-eFluor 710	GIGD7	eBioscience	46-9501-82
CD69	FITC	H1 2F3	BD	553236
Helios	PE	22F6	eBioscience	12-9883-42
CD103	PE-CF594	M290	BD	565849
Gitr	APC	DTA-1	eBioscience	17-5874-81
Pd-1	PerCP-eFluor 710	J43	eBioscience	46-9985-82
Ox40	APC	OX-86	eBioscience	17-1341-82
Klrg1	BV711	2F1	BD	564014
Nrp1	PerCP-eFluor 710	3DS304M	eBioscience	46-3041-82
Icos	APC	C398.4A	eBioscience	17-9949-82
Tbet	PE	4B10	eBioscience	12-5825-82

Gata3	PerCP-eFluor 710	TWAJ	eBioscience	46-9966-42
Ctla-4	APC	UC10-4F10-11	Milipore	MABF545
Ror-γt	BV421	Q31-378	BD	562884
IL-17	PE	TC11-18H1	BD	561020
IFN-γ	PE-CF594	XMG1.2	BD	562303
IL-4	APC	11B11	BD	562045
IL-10	APC	JES5-16E3	Biolegend	505009
IL18Rα	PE	P3TUNYA	eBioscience	12-5183-82
IL-33R	PE	RMST2-2	eBioscience	12-9335-82
CD45	FITC	30.F11	Biolegend	103107
CD31	PE	MEC 13.3	BD	561073
T1α	PE-Cyanine 7	1.1(8.1.1)	BD	25-5381-82
CD326	BV421	G8.8	Biolegend	118225
MHCII	BUV395	2G9	BD	743876
Krt5	Unconjugated<br>Alexa Fluor 488 –<br>Secondary antibody	Poly9059	Biolegend<br>Invitrogen	905901<br>A11039
FR4	PE-Cyanine 7	12A5	eBioscience	11-5445-82
Cell Trace Violet	BV421	N/A	eBioscience	C34571
Live-dead marker	eFluor 506	N/A	eBioscience	65-0866-14
Live-dead marker	Propidium iodide	N/A	eBioscience	BMS500PI

Supplemental Table 1. Flow cytometry reagents.
